# Mitochondrial supercomplex assembly promotes breast and endometrial tumorigenesis by metabolic alterations and enhanced hypoxia tolerance

**DOI:** 10.1038/s41467-019-12124-6

**Published:** 2019-09-11

**Authors:** Kazuhiro Ikeda, Kuniko Horie-Inoue, Takashi Suzuki, Rutsuko Hobo, Norie Nakasato, Satoru Takeda, Satoshi Inoue

**Affiliations:** 1 Division of Gene Regulation and Signal Transduction, Research Center for Genomic Medicine, Saitama Medical University, 1397-1 Yamane, Hidaka-shi, Saitama 350-1241 Japan; 2 Departments of Pathology and Histotechnology, Tohoku University Graduate School of Medicine, 2-1 Seiryo-machi, Aoba-ku, Sendai, 980-8575 Japan; 3 Department of Obstetrics and Gynecology, Saitama Medical Center, Saitama Medical University, 1981, Tsujido, Kamoda, Kawagoe-shi, Saitama, 350-8550 Japan; 4 Department of Obstetrics and Gynecology, Juntendo University, School of Medicine, 2-1-1 Hongo, Bunkyo-ku, Tokyo, 113-0033 Japan; 5 Department of Systems Aging Science and Medicine, Tokyo Metropolitan Institute of Gerontology, 35-2 Sakae-cho, Itabashi-ku, Tokyo, 173-0015 Japan

**Keywords:** Metabolomics, Breast cancer, Endometrial cancer

## Abstract

Recent advance in cancer research sheds light on the contribution of mitochondrial respiration in tumorigenesis, as they efficiently produce ATP and oncogenic metabolites that will facilitate cancer cell growth. Here we show that a stabilizing factor for mitochondrial supercomplex assembly, COX7RP/COX7A2L/SCAF1, is abundantly expressed in clinical breast and endometrial cancers. Moreover, COX7RP overexpression associates with prognosis of breast cancer patients. We demonstrate that COX7RP overexpression in breast and endometrial cancer cells promotes in vitro and in vivo growth, stabilizes mitochondrial supercomplex assembly even in hypoxic states, and increases hypoxia tolerance. Metabolomic analyses reveal that COX7RP overexpression modulates the metabolic profile of cancer cells, particularly the steady-state levels of tricarboxylic acid cycle intermediates. Notably, silencing of each subunit of the 2-oxoglutarate dehydrogenase complex decreases the COX7RP-stimulated cancer cell growth. Our results indicate that COX7RP is a growth-regulatory factor for breast and endometrial cancer cells by regulating metabolic pathways and energy production.

## Abstract (web summary)

Cancer cells rely on mitochondrial respiration to satisfy their metabolic demands. Here the authors show that the mitochondrial supercomplex assembly factor COX7RP is abundantly expressed in breast and endometrial cancer cells and promotes tumor growth and hypoxia tolerance partially by altering levels of the tricarboxylic acid cycle intermediates.

## Introduction

Breast and endometrial cancers are well-known endocrine-related cancers in which estrogen is a stimulant for the initiation and promotion of tumor growth. We have previously identified an estrogen-responsive gene, *COX7RP/COX7A2L/SCAF1*, by genomic binding site cloning and named it after its similarity with cytochrome *c* oxidase subunit 7a^1^. Functional studies performed by us and other groups have shown that COX7RP is a nuclear DNA-encoded mitochondrial gene that can promote respiratory supercomplex assembly, and is critical for mitochondrial respiration and the optimized use of available substrates^2–5^. Furthermore, *Cox7rp*-deficient or *COX7RP*-transgenic mouse models have revealed that this molecule has an important role in muscle activity as well as adaptive thermogenesis in vivo^2^. Today, the physiological relevance of COX7RP in supercomplex formation is widely accepted, although its precise functions in various tissues and diseases remain elusive.

In the biology of rapidly growing tumors, metabolic demand is often elevated, thus a more-efficient energy source is required for continuous growth. Although aerobic glycolysis, or Warburg effect^6,7^, has been assumed as a prototypic characteristics of cancer cells for years, recent cancer studies provide evidence that mitochondrial respiration has important roles in tumorigenesis. For example, various tumor cells including pancreatic cancer^8,9^, breast cancer^10^, and acute myelogenous leukemia^11^ cells have been shown to rely on mitochondrial respiration. In addition, metabolic pathways or processes associated with mitochondria such as glucose metabolism, lipogenesis, amino-acid metabolism, and nucleotide biosynthesis are found to contribute to tumor progression^12^. Not just as a powerhouse for ATP production, mitochondria will generate oncogenic metabolites such as 2-oxoglutarate and succinate, which can alter epigenetic states of cancer cells^13,14^. Mitochondria also generate reactive oxygen species (ROS), which will consequently facilitate DNA mutations and tumor progression^15^.

We question whether COX7RP also contributes to the biology of estrogen-dependent tumors. In the present study, we investigate the clinical relevance and the pathophysiological roles of COX7RP in breast and endometrial cancers. Knockdown and overexpression studies of COX7RP in estrogen-sensitive breast and endometrial cancer cells show that this molecule facilitates cell proliferation and cell cycle promotion, particularly in hypoxic environments, by increasing cytochrome *c* oxidase (COX) activity and mitochondrial energy production. Moreover, COX7RP modulates metabolic pathways in cancer cells. Our results reveal the relevance of mitochondrial respiration in an estrogen-sensitive cancer system, which is particularly enhanced in tumor microenvironments with reduced oxygen availability.

## Results

### Overexpression of COX7RP in breast and endometrial cancers

**Fig. 1:**
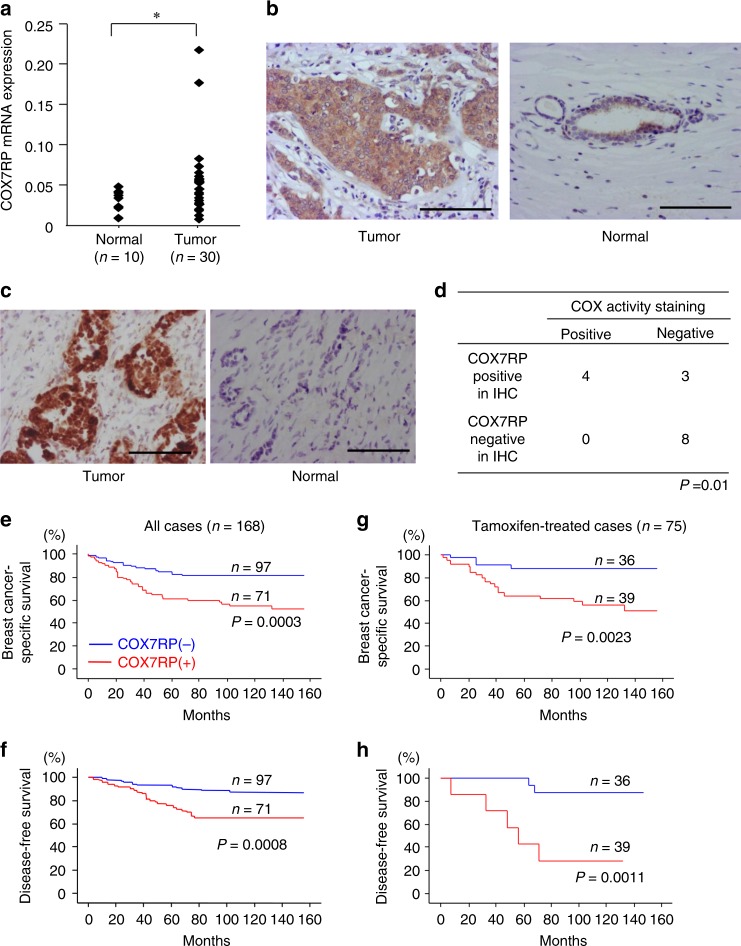
Increased expression of COX7RP in breast cancer. **a** Quantitative RT-PCR analysis of *COX7RP* mRNA expression in breast cancer (*n* = 30 specimens) and normal mammary samples (*n* = 10 specimens). *P < 0.05 for normal versus tumor samples. **b** Sections of human breast cancer tissues and normal mammary glands were immunohistochemically stained for COX7RP. **c** Sections of human breast cancer tissue and normal mammary gland were stained for COX activity. Scale bar, 100 μm. **d** Positive correlation between signals for COX7RP immunohistochemistry and COX activity staining in human breast cancer samples (*n* = 10 specimens). IHC, immunohistochemistry. **e**–**h** Breast cancer-specific survival **e**, **g** and disease-free survival **f**, **h** of clinical breast cancer patients. All cases **e**, **f** and patients with tamoxifen treatment **g**, **h** were analyzed using Kaplan–Meier method

To investigate the clinical relevance of COX7RP in breast cancer, we evaluated *COX7RP* mRNA expression in cancerous and non-cancerous mammary tissues from 40 individuals. The mean level of *COX7RP* mRNA expression in tumors was significantly higher than that in normal regions (Fig. 1a). Next, we performed immunohistochemical staining in tumors and normal tissues using an antibody specific for COX7RP, which is an affinity-purified rabbit IgG generated from the serum of rabbits immunized with the C-terminal 14 amino-acid peptide of COX7RP conjugated with keyhole limpet hemacyanin. In breast tumor tissues with positive COX7RP staining, its immunoreactivity was generally more intense as compared with normal mammary tissues, in which the immunoreactivity was predominantly localized in epithelial cells (Fig. 1b). COX activity in breast cancer tissues was also assessed by COX staining using frozen tissue sections (Fig. 1c). A positive correlation was observed between COX7RP immunoreactivity and COX enzymatic staining (Fig. 1d). In a clinicopathological study evaluating the association between COX7RP immunoreactivity and various variables in 168 breast carcinomas, COX7RP immunoreactivity was significantly associated with lymph node status (*P* = 0.003, *χ*^2^ test), ERα status (*P* = 0.01, *χ*^2^ test), or ERα-labeling index (*P* = 0.0004, *χ*^2^test) (Supplementary Table 1). Subjects with COX7RP-positive tumors showed significantly poorer disease-free and breast cancer-specific survivals than those with COX7RP-negative tumors (Fig. 1e, f). Moreover, this correlation was also observed in breast cancer patients treated with tamoxifen (Fig. 1g, h). Univariate analysis indicated that factors including lymph node status, HER2 status, histological grade, COX7RP immunoreactivity, and ERα status were correlated with poor breast cancer-specific survival (Supplementary Table 2). A multivariate analysis revealed that lymph node status, histological grade, COX7RP immunoreactivity, and ERα status were independent prognostic factors with *P* values < 0.05 (Cox proportional hazard model).

We also investigated COX7RP expression in clinical endometrial cancer samples. Substantial COX7RP immunoreactivities were detected in endometrial tumors (Supplementary Fig. 1a), whereas rather weak immunoreactivities were observed in normal glandular epithelial regions (Supplementary Fig. 1b). Some of the tumor samples exhibited higher *COX7RP* mRNA levels and the mean levels were significantly higher in tumors than in normal regions (Supplementary Fig. 1c). These results indicate that COX7RP expression could have clinical relevance in estrogen-related breast and endometrial cancers.

### COX7RP promotes breast and endometrial cancer growth

**Fig. 2:**
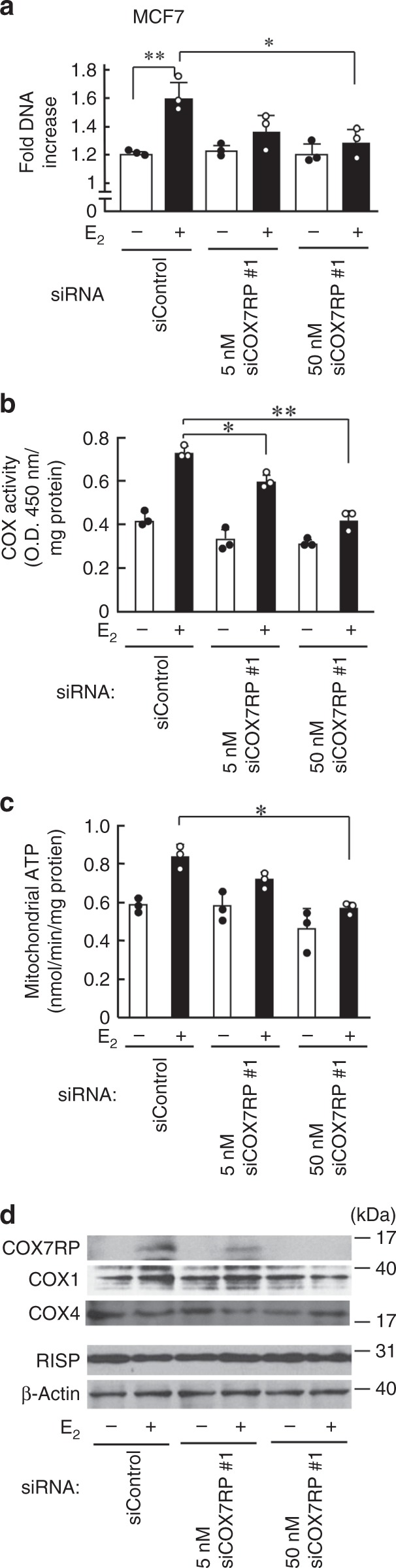
Inhibition of COX7RP expression suppresses estrogen-induced cell proliferation and ATP production in MCF7 cells. **a** siCOX7RP attenuates hormone-dependent growth of MCF7 cells. MCF7 cells were transfected with siCOX7RP or siControl for the indicated times and cell growth was estimated by the DNA amount. **b** Silencing of COX7RP decreases COX activity. COX activity was assessed in MCF7 cells treated with siCOX7RP or siControl. **c** Silencing of COX7RP decreases ATP production. ATP production in mitochondria was assessed in MCF7 cells treated with siCOX7RP or siControl. Data are presented as means ± SD (*n* = 3 independent experiments). *P < 0.05, **P < 0.01, Student’s *t* test. **d** siCOX7RP attenuates estrogen-induced COX7RP expression. Cell lysates were subjected to western blotting using antibodies for COX7RP, COX1, COX4, RISP, and β-actin. Source data are provided as a Source Data file

We next asked whether COX7RP contributes to estrogen-mediated breast cancer growth. As a first step, we investigated the role of COX7RP in estrogen-dependent growth of MCF7 cells. Western blot analysis revealed that COX7RP protein levels were upregulated by 17β-estradiol (E_2_) and substantially repressed by a COX7RP-specific siRNA (siCOX7RP #1) in cultured MCF7 cells (Fig. 2a, d). DNA synthesis increased following the treatment of MCF7 cells with E_2,_ whereas siCOX7RP #1 significantly repressed the estrogen-dependent DNA increase. Estrogen treatment also increased COX activity (Fig. 2b) and mitochondrial ATP synthesis (Fig. 2c) in MCF7 cells, both of which were repressed by siCOX7RP #1 or by another siRNA targeting COX7RP (siCOX7RP #2) (Supplementary Fig. 2). In terms of subunits for mitochondrial respiratory complex IV (COX1 and COX4) and complex III (Rieske iron–sulfur protein, RISP), estrogen did not show apparent influence on the protein levels of these subunits (Fig. 2d). These siRNAs targeted COX7RP also repressed COX activity and mitochondrial ATP synthesis in endometrial cancer Ishikawa cells. Namely, estrogen increased COX7RP protein expression and growth and siCOX7RP #1 decreased estrogen-dependent growth (Supplementary Fig. 3a). Estrogen treatment also increased COX activity (Supplementary Fig. 3b) and mitochondrial ATP synthesis (Supplementary Fig. 3c) in Ishikawa cells, both of which were repressed by siCOX7RP #1. siCOX7RP #2 also impaired the estrogen-dependent increase in tumor growth, COX activity, and mitochondrial ATP synthesis in Ishikawa cells (Supplementary Fig. 4). These results indicate that COX7RP stimulates the growth of MCF7 and Ishikawa cells through the upregulation of COX activity and ATP production.

**Fig. 3:**
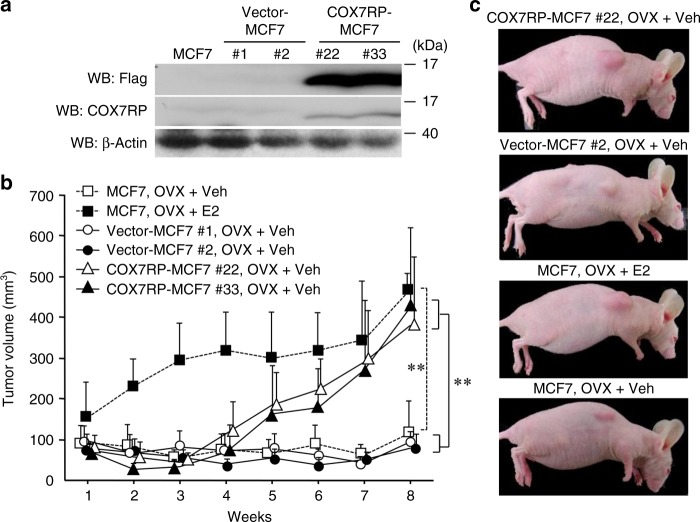
COX7RP promotes estrogen-independent tumor growth of MCF7 cells. **a** Generation of MCF7 cells stably expressing COX7RP (COX7RP-MCF7 #22 and #33) or control vector (vector-MCF7 #1 and #2). MCF7 cells were transfected with Flag-tagged COX7RP or empty vector and selected in medium containing G418. Western blot analysis was performed with anti-Flag, anti-COX7RP, and β-actin antibodies. **b**, **c** Tumor growth without gonadal estrogen was observed in ovariectomized (OVX) nude mice bearing COX7RP-MCF7 cells. Ovariectomized athymic mice were inoculated with MCF7-derived cells and administrated with or without 1 μg/kg E_2_ or vehicle (Veh) twice a week for 8 weeks, and the tumor volumes (mm^3^) were calculated. Data are presented as means ± SD (*n* = 5 animals). **, *P* < 0.01 for MCF7 with OVX + vehicle versus MCF7 with OVX + E_2_, and vector-MCF7 #1 and #2 with OVX + Veh versus COX7RP-MCF7 #22 and #33 with OVX + Veh (two-way analysis of variance). Source data are provided as a Source Data file

Considering that the elevation of COX7RP expression was implicated in the acceleration of breast cancer cell growth, we further tested whether COX7RP overexpression was sufficient to promote the proliferation of cancer cells in vivo. For this purpose, stable MCF7 cell lines ectopically expressing FLAG-tagged COX7RP (COX7RP-MCF7 #22 and #33) or FLAG expression vector (vector-MCF7 #1 and #2) were generated (Fig. 3a). COX7RP-MCF7 cells showed an increase in COX activity and ATP production in mitochondria (Supplementary Fig. 5). We implanted parental MCF7 cells, vector-transfected, or COX7RP-expressing MCF7 cells into ovariectomized female athymic mice. Mice with parental MCF7 cells were treated with E_2_ or vehicle, and mice with MCF7 transfectants were treated with vehicle alone. The tumor volume in mice bearing COX7RP-MCF7 cells was significantly larger as compared with mice bearing vector-MCF7 cells, almost reaching the volume of parental MCF7 cell-derived tumors treated with E_2_ by 8 weeks (Fig. 3b, c). Thus, COX7RP-MCF7 cells acquired an ability to grow independent of estrogen.

**Fig. 4:**
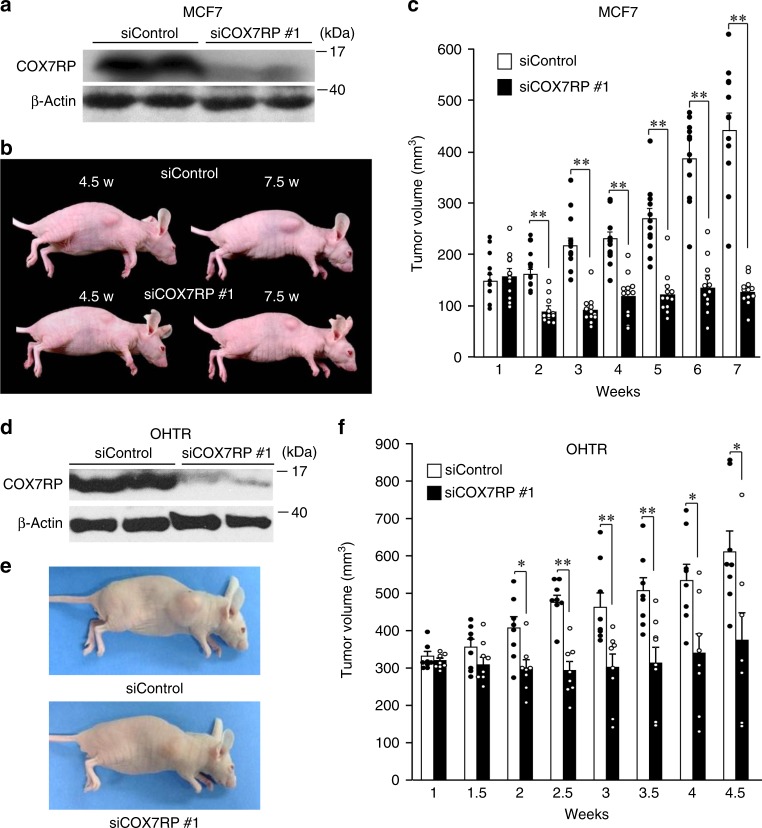
Inhibition of COX7RP expression suppresses tumor growth of parental MCF7 cells and MCF7-derived tamoxifen-resistant cells. **a** siCOX7RP decreases COX7RP protein expression in tumors obtained from mice inoculated with MCF7 cells. Athymic female mice were inoculated with 10 million MCF7 cells and then administrated with control siRNA or COX7RP siRNA (5 μg twice a week). COX7RP protein expression was decreased in MCF7-derived tumors treated with siCOX7RP. Western blot analysis was performed for MCF7-derived tumors treated with siControl or siCOX7RP for 7.5 weeks. **b** Reduced tumor size in mice 4.5 or 7.5 weeks after inoculation with siControl or siCOX7RP. **c** siCOX7RP decreases tumor growth of MCF7 cells in athymic mice. Volume (mm^3^) of tumors caused by MCF7 cells in athymic mice. Data are presented as means ± SEM. *P < 0.05; **P < 0.01, Student’s *t* test; *n* = 12 animals. **d** siCOX7RP decreases COX7RP protein expression in tumors obtained from mice inoculated with MCF7-derived 4-hydroxytamoxifen-resistant (OHTR) cells. Athymic female mice were inoculated with 10 million OHTR cells and then administrated with control siRNA or COX7RP siRNA (5 μg twice a week). COX7RP protein expression was decreased in OHTR-derived tumors treated with siCOX7RP. Western blot analysis was performed for OHTR-derived tumors treated with siControl or siCOX7RP. **e** Reduced tumor size in mice 4.5 weeks after inoculation with control siRNA or COX7RP siRNA. **f** siCOX7RP decreases tumor growth of OHTR cells in athymic mice. Volume (mm^3^) of tumors caused by MCF7 cells in athymic mice. Data are presented as means ± SEM. *P < 0.05; **P < 0.01, Student’s *t* test; *n* = 8 animals. Source data are provided as a Source Data file

Subsequently, we determined the effect of COX7RP knockdown on MCF7 tumor growth in a xenograft model using athymic mice. MCF7 cells were implanted into the flanks of athymic female mice and then they were injected with siCOX7RP #1 or control siRNA (siControl). Western blot analysis showed that COX7RP protein levels were substantially reduced in MCF7 cell-derived tumors obtained from athymic mice continuously treated with siCOX7RP #1 (Fig. 4a). Notably, tumor growth was significantly reduced in mice treated with siCOX7RP #1, as compared with mice treated with siControl (Fig. 4b, c). siCOX7RP #1 was also used in the xenograft model with tamoxifen-resistant breast cancer cells (OHTR), which were established from parental MCF7 cells by long-term 4-hydroxytamoxifen (OHT) treatment. The siCOX7RP #1 injection decreased COX7RP protein levels (Fig. 4d) and tumor formation (Fig. 4e, f) of OHTR cell-derived tumors as compared with mice with siControl. Similarly, we generated Ishikawa cells stably expressing COX7RP (COX7RP-Ishikawa #1 and #28) and vector (vector-Ishikawa #9 and #10) (Supplementary Fig. 6a). COX7RP overexpression elevated COX activity and ATP production in Ishikawa cells (Supplementary Fig. 7a, b). COX7RP-Ishikawa cells formed significantly larger tumors in athymic female mice than vector-Ishikawa cells (Supplementary Fig. 6b, c). siCOX7RP #1 decreased COX7RP protein levels in tumor tissues and decreased tumor formation of the Ishikawa cell-derived xenograft model using athymic mice (Supplementary Fig. 8).

### COX7RP promotes hypoxia resistance

**Fig. 5:**
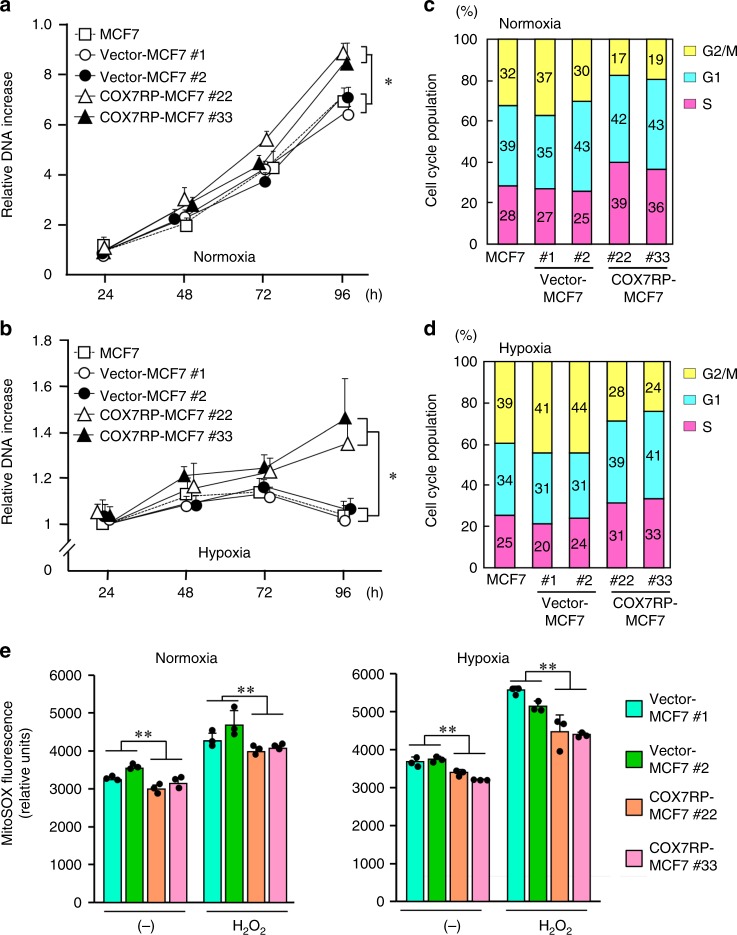
Overexpression of COX7RP induces hypoxia tolerance and decreases mitochondrial ROS. **a** Growth of COX7RP-MCF7 cells was promoted in normoxic culture conditions (20% O_2_). **b** Continual growth of COX7RP-MCF7 cells in hypoxic culture conditions (1% O_2_). COX7RP overexpression increases the percentage of MCF7 cells in the proliferation stage of the cell cycle in normoxia **c** and hypoxia **d**. **e** Decrease in mitochondrial ROS levels in COX7RP-MCF7 cells. Cells were exposed to hypoxia (1% O_2_) in the presence or absence of H_2_O_2_ (1 mm) for 8 h, subsequently incubated with mitochondrial ROS probe MitoSOX. Fluorescence intensity was measured by a fluorimetry assay. Data are presented as means ± SD (*n* = 3 independent experiments). *P < 0.05; **P < 0.01, Two-way analysis of variance. Source data are provided as a Source Data file

We explored the role of COX7RP on cancer cell growth in hypoxic states. Cell growth, assessed as the amount of DNA isolated, was promoted in COX7RP-MCF7 cells as compared with vector-MCF7 or parental MCF7 cells in a 20% O_2_ environment after 96 h (Fig. 5a), and this phenomenon was further emphasized in a 1% O_2_ hypoxic condition (Fig. 5b), rather than in mild hypoxic condition of 5–10% O_2_ concentrations (Supplementary Fig. 9a). COX7RP overexpression increased the fraction of cells in S phase in both normoxic and hypoxic states (Fig. 5c, d). COX7RP also inhibited the hypoxia-induced generation of mitochondrial ROS, as shown by the mitochondrial ROS-specific probe MitoSOX (Fig. 5e). In Ishikawa cells, under a hypoxic condition (1% O_2_), COX7RP-Ishikawa cells continued to grow after 96 h, wheresas vector-transfected and parental cells could not (Supplementary Fig. 9b, 10a, b). Furthermore, COX7RP-Ishikawa cells showed a high percentage of cells in S phase in both normoxia and hypoxia as compared with control cells (Supplementary Fig. 10c, d), but maintained a lower ROS level (Supplementary Fig. 10e). These results show that COX7RP promotes the growth of estrogen-dependent breast cancer cells and increases their resistance to hypoxia. Mitochondrial DNA amounts in MCF7 and Ishikawa cells stably expressing COX7RP were not substantially different from those in their corresponding vector-transfected clones, respectively, implying that COX7RP overexpression does not basically affect mitochondrial quantity (Supplementary Fig. 11).

### COX7RP induces respiratory supercomplex assembly in hypoxia

**Fig. 6:**
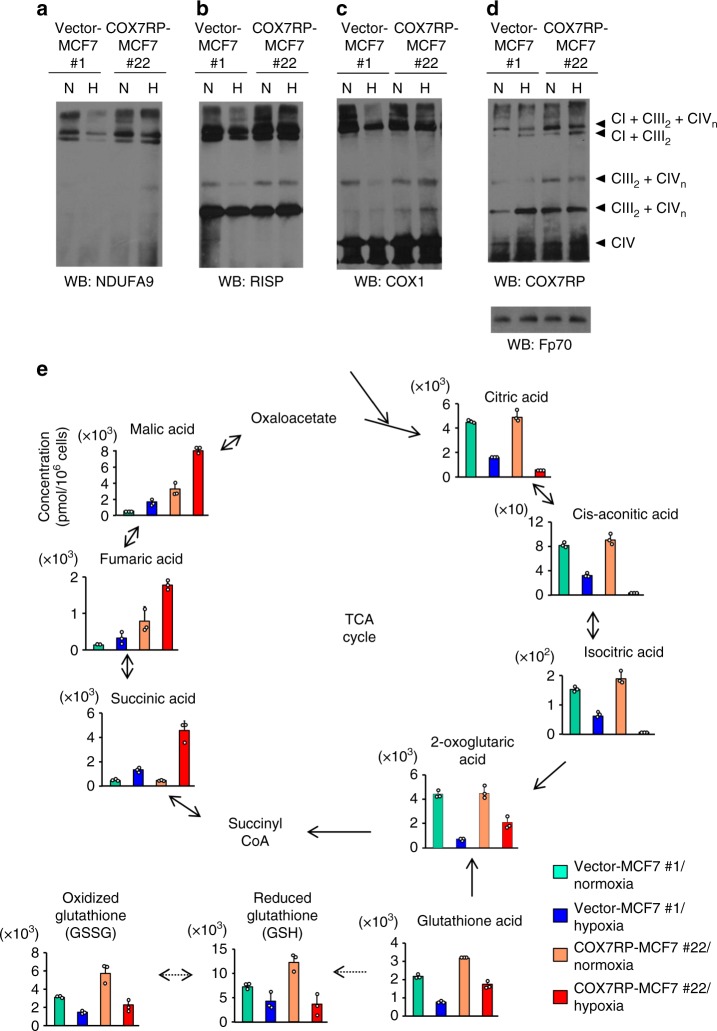
COX7RP facilitates supercomplex assembly in hypoxia and modulates metabolic pathways. **a**–**d** Mitochondrial proteins of COX7RP-MCF7 cells exposed to normoxic (N) and hypoxic (H: 1% O_2_) conditions were solubilized with digitonin (4 g g^−1^) and subjected to BN-PAGE. Western blot analysis was performed with antibodies for NDUFA9 **a**, RISP **b**, COX1 **c**, COX7RP **d**, and Fp70 **e**. Positions corresponding to CI + CIII_2_ + CIV_n_, CIII_2_ + CIV_n_, CIII_2_ or CIV_n_, and CIV are indicated. **e** COX7RP-MCF7 cells were cultured in normoxic or hypoxic conditions (1% O_2_) for 24 h and cell extracts were subjected to metabolome analysis using capillary electrophoresis time-of-flight mass spectrometry (CE-TOFMS) for cation analysis and capillary electrophoresis-tandem mass spectrometry (CE-MS/MS) for anion analysis. Metabolites in the TCA cycle are schematically shown. The intracellular concentration (pmol/million cells) of key metabolites involved in the TCA cycle and glutathione synthesis pathway are shown as means ± SD (*n* = 3 biologically independent samples). Source data are provided as a Source Data file

We next investigated the contribution of COX7RP to supercomplex assembly during hypoxia. Mitochondria were prepared from COX7RP-MCF7 #22 and vector-MCF7 cells #1 cultured in normoxic or hypoxic conditions and analyzed by blue native polyacrylamide gel electrophoresis (BN-PAGE) with subsequent immunoblotting. The results showed that hypoxia decreased the signal for the complex I (CI) subunit NDUFA9 at the position of supercomplex (CI + CIII_2_ + CIV_n_) in vector-MCF7 cells #1 as compared with the signal in the cells under normoxic condition (Fig. 6a and Supplementary Table 3). The NDUFA9 signals at the position of supercomplex (CI + CIII_2_ + CIV_n_) in both normoxia and hypoxia were increased in COX7RP-MCF7 #22 cells as compared with those in vector-MCF7 #1 cells (Fig. 6a). Slight decrease of NDUFA9 signal at the position of supercomplex (CI + CIII_2_) was observed in hypoxia in vector-MCF7 #1 cells. Signals for RISP, a CIII late stage subunit of the supercomplex^16^, were increased at the position of supercomplex (CIII_2_ + CIV_n_) in COX7RP-MCF7 #22 cells as compared with vector-MCF7 #1 cells (Fig. 6b). The COX1 signal was also increased at the position of supercomplex (CIII_2_ + CIV_n_) in COX7RP-MCF7 #22 cells under hypoxic condition as compared with the signal in vector-MCF7 #1 cells (Fig. 6c), whereas the COX1 signals at the position of CIV were not significantly different between the two clones (Fig. 6c and Supplementary Table 3). Probing the blots with an anti-COX7RP antibody revealed that COX7RP protein was more abundantly detected in the positions at supercomplexes (CI + CIII_2_ + CIV_n_ and CIII_2_ + CIV_n_) during hypoxia in COX7RP-MCF7 #22 cells (Fig. 6d and Supplementary Table 4). We next investigated the supercomplex assembly in OHTR cells. Mitochondria were prepared from OHTR cells and its parental MCF7 cells, which were cultured in the presence or absence of OHT, and analyzed by BN-PAGE with subsequent immunoblotting (Supplementary Fig. 12). Notably, signals for CI + CIII_2_ + CIV_n_ and CIII_2_ + CIV_n_ were increased in OHTR cells even in the presence of tamoxifen as compared with MCF7 cells (Supplementary Table 5). In COX7RP-Ishikawa #28 cells, signals for NDUFA9, RISP, and COX7RP were also increased at the positions of supercomplex (CI + CIII_2_ + CIV_n_ and CIII_2_ + CIV_n_) as compared with the corresponding vector-transfected cells (Supplementary Fig. 13a–c, and Supplementary Tables 6 and 7). These results suggest that the relevance of COX7RP in supercomplex assembly would be critical in hypoxic states as well as in normoxic states.

### COX7RP modulates metabolism

We next examined metabolomic profiles of COX7RP-MCF7 #22 and vector-MCF7 #1 cells in normoxia or hypoxia, analyzing by the steady-state levels of 116 metabolites based on capillary electrophoresis time-of-flight mass spectrometry (CE-TOFMS) (Fig. 6e; Supplementary data 1). We noted that the levels of TCA cycle metabolites including succinic acid, fumaric acid, malic acid, and 2-oxoglutaric acid were all elevated in hypoxia-cultured COX7RP-MCF7 cells. In contrast, the levels of citric acid, cis-aconitic acid, and isocitric acid decreased during hypoxia. In addition, the concentration of glutamic acid and total glutathione (GSSG + GSH) increased in COX7RP-overexpressing cells. The amounts of 2-oxoglutaric acid and succinic acid were further verified in additional stable clones of MCF7 cells (Vector-MCF7 #2 and COX7RP-MCF7 #33) (Supplementary Fig. 14a, c). In terms of NAD(H), the total levels were substantially increased in COX7RP-expressing cells compared with control cells based on metabolomics analysis (Supplementary Data 1). It is also notable that mitochondrial NAD(H) levels were increased in COX7RP-expressing MCF7 cells, suggesting that COX7RP induces a metabolic change to increase the mitochondrial NAD(H) level, which would facilitate the growth of MCF7 cells in hypoxia (Supplementary Fig. 14e).

To further dissect the metabolic regulation, we performed tracing experiment with ^13^C_5_-glutamine in MCF7 clones in both normoxia and hypoxia (Supplmentary Fig. 15, Supplementary data 2). The result showed that the production of ^13^C_4_-labeled succinic acid and malic acid was activated in COX7RP-expressing cells compared with control cells in hypoxia, suggesting that it was originated from ^13^C_5_-glutamine via subsequent ^13^C_5_-labeled 2-oxoglutatic acid using the half part of TCA cycle. It is also notable that the percentages of ^13^C_5_-labeled isocitric acid, cis-aconitic acid, and citrate were increased after ^13^C_5_-glutamine labeling, indicating a reductive carboxylation from glutamine to citrate.

**Fig. 7:**
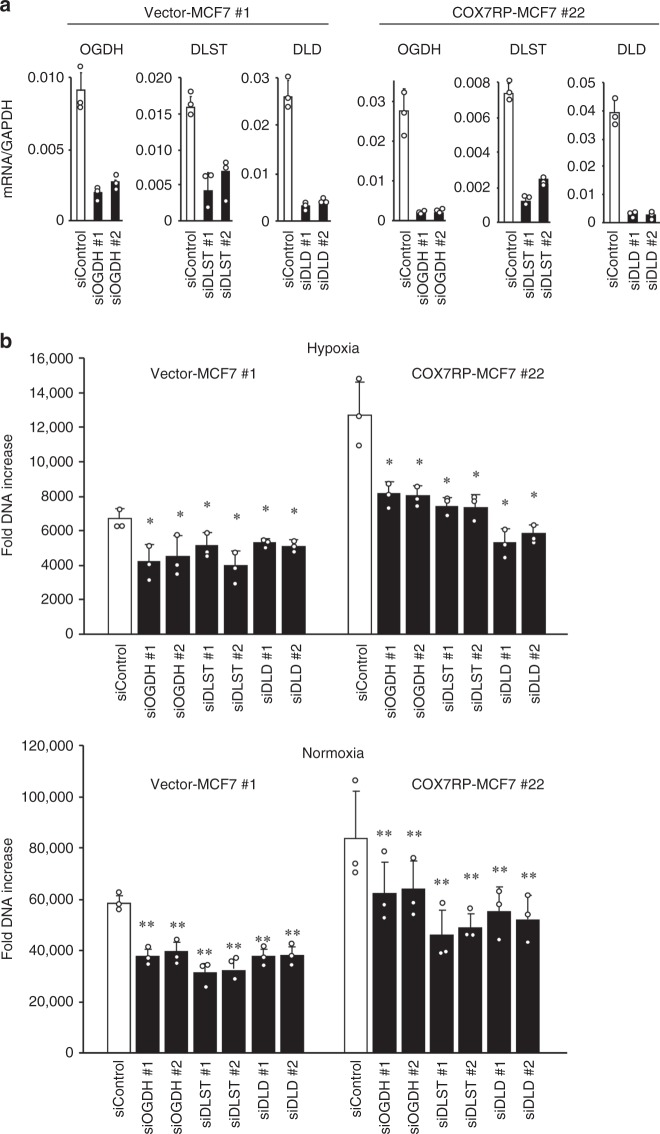
Silencing of 2-oxoglutarate dehydrogenase suppresses MCF7 cell growth. **a** Effects of siRNAs on mRNA expression of 2-oxoglutarate dehydrogenase. siRNAs targeting each subunit of the 2-oxoglutarate dehydrogenase complex (*OGDH*, *DLST*, and *DLD*) were transfected into COX7RP-MCF7 and vector-MCF7 cells. Quantitative RT-PCR analysis of mRNA expression was performed. **b** Effects of siRNAs on the growth of vector-MCF7 and COX7RP-MCF7 cells under hypoxic (1% O_2_) or normoxic conditions. Cell growth was estimated by DNA assay. Data are presented as means ± SD (*n* = 3 independent experiments). *P < 0.05, **P < 0.01, Student’s *t* test. Source data are provided as a Source Data file

Transcriptomic analysis also revealed that a number of metabolism-associated genes were upregulated in COX7RP-overexpressing cells as compared with vector control cells in hypoxia (Supplementary Table 8). In addition, we examined the mRNA levels of key regulatory genes/factors involved in the TCA cycle and malate dehydrogenases (Supplementary Fig. 16a). Basically, the genes coding for the 2-oxoglutarate dehydrogenase complex (DLD, DLST, and OGDH) and malate dehydrogenase (MDH1 and MDH2) were upregulated in COX7R-MCF7 cells as compared with vector-MCF7 cells in hypoxia. Because the steady-state levels of succinic acid, fumaric acid, and malic acid increased in COX7RP-MCF7 cells during hypoxia (Fig. 6e), we further focused on the role of the 2-oxoglutarate dehydrogenase complex in cancer cell growth. This complex is a TCA cycle enzyme that decarboxylates 2-oxoglutarate to succinyl-CoA. The siRNA-mediated knockdown of its subunits, DLD, DLST, and OGDH (Fig. 7a), substantially repressed the growth of COX7RP-overexpressing cancer cells in both hypoxic and normoxic conditions (Fig. 7b). In comparison with 2-oxoglutarate dehydrogenase, silencing of malate dehydrogenase subunits (MDH1 and MDH2) did not dampen the growth of COX7RP-MCF7 and vector-MCF7 cells in hypoxia and normoxia (Supplementary Fig. 17).

Besides, we evaluated mRNA expression of GSH-synthesizing enzymes *GCLM* and *GSS* and found that mRNA levels of these enzymes were significantly increased in COX7RP-MCF7 cells compared with control cells (Supplementary Fig. 18), suggesting that glutathione levels may be elevated by the upregulation of GSH-synthesizing enzymes. These results indicate that COX7RP could be a critical modulator of metabolic pathways in MCF7 cells. Furthermore, comparison between metabolomic profiles of COX7RP-Ishikawa #28 and vector-Ishikawa #9 cells in normoxia or hypoxia exhibited the similar regulation of TCA cycle metabolites as in MCF7 transfectants (Supplementary Fig. 13d). Studies using additional stable clones of Ishikawa (Vector-Ishikawa #10 and COX7RP-Ishikawa #1) also showed the elevation of cellular levels of 2-oxoglutaric acid and succinic acid as well as of mitochondrial NAD(H) levels (Supplementary Fig. 14b, d, f). The gene expression of 2-oxoglutarate dehydrogenase complex (DLD, DLST, and OGDH) and malate dehydrogenase (MDH1 and MDH2) were also upregulated in COX7RP-Ishikawa cells as compared with vector-Ishikawa cells in hypoxia (Supplementary Fig. 16b). Silencing of 2-oxoglutarate dehydrogenase subunits also inhibited the growth of COX7RP- and vector-Ishikawa cells during hypoxia and normoxia (Supplementary Fig. 19).

Although the growth of COX7RP-expressing cells was significantly increased compared with that of control cells in hypoxia (Fig. 5 and Supplementary Fig. 10), we showed that the DNA contents of control cells also increased 4 days after the supplementation of either pyruvate or cell-permeable succinate to almost the same levels as COX7RP-expressing cells, suggesting that both permeable succinate and pyruvate could be utilized as respiration substrates that improve energy production (Supplementary Fig. 20).

### COX7RP stimulates oxygen consumption in hypoxia

**Fig. 8:**
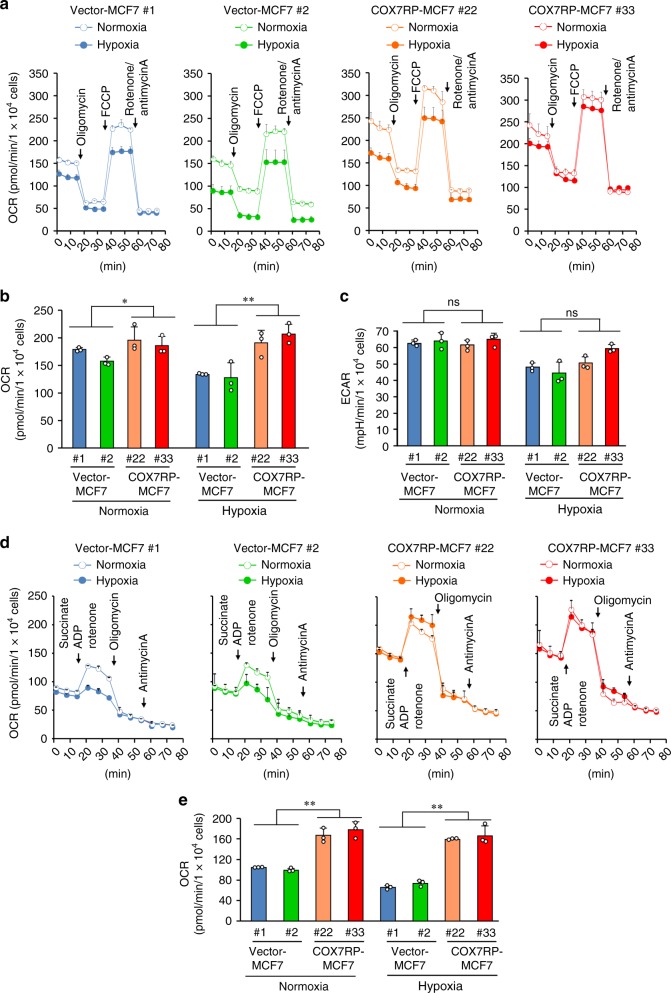
Overexpression of COX7RP promotes oxygen consumption after hypoxia-mimetic treatment. **a**–**c** CI-mediated respiration. COX7RP-MCF7 and vector-MCF7 cells were incubated overnight in normoxic or hypoxic (1% O_2_) conditions. The medium was then replaced with serum-free XF medium containing glucose and sodium pyruvate as substrates. Mitochondrial stress test was performed at 37 °C on a Seahorse Bioscience XF Extracellular Flux Analyzer using a Cell Mito Stress Test Kit and oxygen consumption rates (OCRs) were plotted **a**. Maximal OCRs **b** and basal extracellular acidification rate (ECAR) **c** are presented as means ± SD (*n* = 3 biologically independent samples). **d**, **e** CII-mediated respiration. Mitochondrial stress test was performed as described above using succinate as a substrate in the presence of rotenone **d**. OCRs are presented as means ± SD (*n* = 3 biologically independent samples). Maximal OCRs **e** were measured as the oxygen consumption rate after FCCP addition minus the non-mitochondrial respiration. *P < 0.05; **P < 0.01, two-way analysis of variance. Source data are provided as a Source Data file

To assess the effect of COX7RP on cellular bioenergetics under hypoxic conditions, extracellular flux analysis was performed to determine the oxygen consumption rate (OCR) and extracellular acidification rate (ECAR), which are assumed to reflect mitochondrial oxidative phosphorylation activity and lactate production that can be equated to glycolytic activity, respectively^17^. COX7RP-MCF7 and vector-MCF7 clones were cultured in normoxic (20% O_2_) or hypoxic condition (1% O_2_). We measured the OCR and ECAR using substrates for complex I (glucose and pyruvate) or complex II (succinate) by a Seahorse Bioenergizer (Seahorse Bioscience, Agilent). Complex I substrate-based cellular OCR was substantially elevated in COX7RP-MCF7 cells as compared with vector-MCF7 cells in both normoxic and hypoxic conditions (Fig. 8a). The maximum respiration rate was significantly increased in COX7RP-MCF7 clones as compared with vector-MCF7 clones (Fig. 8b). On the other hand, the ECAR did not basically differ between COX7RP-MCF7 and vector-MCF7 clones (Fig. 8c). Intriguingly, complex II substrate-based OCR could also be enhanced in COX7RP-overexpressing MCF7 cells as compared with vector control cells in both normoxic and hypoxic conditions (Fig. 8d, e). In contrast, either COX7RP or OGDH knockdown by siRNAs decreased the maximum respiration rate in MCF7 cells (Supplementary Fig. 21).

## Discussion

In the present study, we found that mitochondrial respiratory supercomplex assembly-stabilizing factor COX7RP is overexpressed in clinical breast and endometrial cancers. Cancer cell experiments demonstrated that COX7RP overexpression promotes breast and endometrial cancer cell growth and stabilizes supercomplex assembly even in hypoxia. Metabolomic analysis reveals that COX7RP overexpression modulates the metabolic profile of cancer cells, particularly the steady-state levels of TCA cycle intermediates. Silencing of 2-oxoglutarate dehydrogenase complex subunits decreased the COX7RP-stimulated cancer cell growth. Overexpression of COX7RP increased oxygen consumption in breast cancer cells. Moreover, COX7RP immunoreactivity associates with prognosis of breast cancer patients, and also correlates with the poor prognosis of tamoxifen-treated breast cancer patients. Our results propose that COX7RP is a growth-regulatory factor for estrogen-sensitive cancer cells that regulates metabolic pathways and energy production.

Enriquez and his colleagues recently proposed that COX7RP/SCAF1 is required for the assembly of the CIII_2_ + CIV supercomplex structure^18^. COX7RP promotes the interaction between CIII and CIV in both CI + CIII_2_ + CIV_n_ and CIII_2_ + CIV_n_. Sazanov and his colleagues also proposed that there are two distinct arrangements of tight and loose forms of supercomplexes that include CIV^19^. COX7RP co-migrates with most supercomplexes containing CIV and may promote the stability of the supercomplexes^19^. In the present study, we found that COX7RP overexpression stimulates the assembly of supercomplexes CI + CIII_2_ + CIV_n_ and CIII_2_ + CIV_n_ even in hypoxia and that the COX7RP protein co-migrates with the supercomplexes CI + CIII_2_ + CIV_n_ and CIII_2_ + CIV_n_.

As complex IV levels shown by COX1 immunoblots were not significantly altered by COX7RP overexpression, we assume that the increase in COX activity in COX7RP-overxpressing cells is likely originated from enhanced levels of CI + CIII_2_ + CIV_n_ and CIII_2_ + CIV_n_. Notably, CI + CIII_2_ + CIV_n_ levels shown by NDUFA9 were reduced by hypoxia but relatively higher in COX7RP-overexpressing cells than control cells. NDUFA9 is required at a late assembly step critical for complex I biogenesis and activity^20,21^. Complex I is known to exist in either active or deactive states according to its catalytic status and complex I converts spontaneously to its deactive state under hypoxic conditions^22^. Notably, a recent structure study of deactive state of mammalian complex I showed that the loss of ordered structures around the ubiquinone-binding site including NDUFA9 is characteristic of the deactive state of complex I^23^. We thus assume that complex I probed for NDUFA9 might indicate relative abundance of active state of complex I, which may be repressed by hypoxia but relatively maintained in COX7RP-overexpressing cells. Our data would show a possibility that COX7RP overexpression may modulate the complex I activity. These results suggest that COX7RP has an important role in the efficient assembly of mitochondrial respiratory supercomplexes and provides resistance to hypoxia in estrogen-sensitive breast and endometrial cancer cells.

Interestingly, it has been reported previously that supercomplex formation could modulate the production of mitochondrial ROS, which was principally generated at the electron transport chain during the process of oxidative phosphorylation (OXPHOS)^24^. Indeed, Maranzana et al. demonstrated that loss of supercomplex organization causes a marked enhancement of ROS generation by complex I^25,26^. Mitochondrial ROS production is usually increased along with the activation of mitochondrial respiration, as exemplified by a study in chemotherapy-resistant breast cancer stem cells^27^. We thus assume that enhanced supercomplex formation by COX7RP overexpression may repress ROS production depending on the efficiency of electron transfer among complexes I, III, and IV. Jang and Javadov recently reported that the knockdown of complex I and II subunits in cardiac mitochondria enhanced ROS production, whereas impaired respirasome formation and ATP production^28^. The supercomplex-mediated ROS inhibition would be an important function of supercomplex assembly in mitochondrial respiration since Fedor and Hirst recently reported that substrate channeling in supercomplexes does not occur^29^. We hypothesize that the physiological relevance of COX7RP may drive to increase its involvement in (1) the reduction of ROS generation as we showed in Fig. 5e and Supplementary Fig. 10e, (2) the improvement of assembly or stability of supercomplexes as we showed in Fig. 6, and Supplementary Figs. 12 and 13, (3) the protection against non-specific aggregation in the high-protein concentration of the mitochondrial inner membrane by enhancing weak interactions between mitochondrial complexes, (4) the promotion of quinone diffusion in the protein-dense membrane by supercomplex formation. In regard to quinone diffusion in the mitochondrial inner membrane^30^, we assume that COX7RP would facilitate to minimize the distance between quinone-binding site in CI and quinol-binding site in CIII in the protein-dense mitochondrial membrane by forming loosely associated but closely packed assemblies, particularly CI + CIII_2_ + CIV_n_. COX7RP-mediated promotion of supercomplex assembly may also protect free quinone diffusion between CI and CIII using CI as a fender around the interface of complexes. Moreover, it is possible that COX7RP-mediated promotion of supercomplex assembly would contribute to a homogenous mixture and distribution of the complexes in the mitochondrial membrane.

Several lines of evidence indicate that the levels of succinate, fumarate, and malate are elevated in cancers as compared with their corresponding normal tissues^31,32^. Succinate and fumarate are known to exhibit oncogenic effects in cancers through epigenetic modification and activation of hypoxic signaling. This occurs because these compounds, which are structurally similar to 2-oxoglutarate, can inhibit the 2-oxoglutarate-dependent dioxygenases^33^. It is possible that COX7RP overexpression stimulates cancer cell growth by the upregulation of these compounds in MCF7 and Ishikawa cells. Moreover, COX7RP overexpression elevated the expression of TCA cycle enzymes during hypoxia, which will facilitate TCA cycle metabolism. Thus, silencing of 2-oxoglutarate dehydrogenase subunits impaired the increase in cancer cell growth by COX7RP overexpression. It is noted that 2-oxoglutarate dehydrogenase complexes have an important role in cell growth in several cancer cells^34,35^. We speculate that COX7RP overexpression may lead to a more active energy flux status by upregulating mitochondrial respiratory supercomplex assembly in hypoxia and thus maintaining the moderate expression levels of these genes. Andrzejewski et al.^36^ recently showed that PPARG coactivator 1α (PGC-1α), which functions as a regulator for mitochondrial respiration, promotes breast cancer metastasis by stimulating global bioenergetic capacity and provides resistance to bioenergetics drugs. PGC-1α-mediated mitochondrial regulation is also involved in endometrial cancer^37^. It was reported that citrate synthase activity, mitochondrial DNA content and expression levels of mitochondria-regulating factors including PGC-1α and mitochondrial transcription factor A are increased especially in estrogen-related type I endometrial cancer^38^. These notions suggest that COX7RP cooperatively modulates energy flux with other mitochondrial factors in hormone-dependent cancers.

In addition to enhanced aerobic glycolysis, cancer cells display an altered glutamine metabolism, which plays important roles in cell proliferation and survival^39^. Hydrolysis of glutamine generates glutamic acid, which is subsequently deaminated to the TCA cycle intermediate, 2-oxoglutaric acid. This pathway is important to replenish the TCA cycle intermediate in rapidly growing cancer cells, as citric acid is used to generate acetyl-CoA for lipid synthesis in the cytosol^40^. As a result of this metabolic shift, the TCA cycle is compensated by contributions from glutamine^41^. Our tracing experiment with ^13^C_5_-glutamine further showed that the production of ^13^C_4_-labeled succinate and malate, which are originated from ^13^C_5_-glutamine via subsequent ^13^C_5_-labeled 2-oxoglutatic acid using the half part of TCA cycle, was activated in COX7RP-expressing cells compared with control cells in hypoxia. The data may indicate that COX7RP overexpression will facilitate succinate-fueled mitochondrial respiration in cancer cells as consistent with the upregulation of OCR. It is notable that isocitrate dehydrogenase-catalyzed cytosolic reductive carboxylation from glutamine to citrate in cells with mitochondrial dysfunction may partly contribute to energy production in COX7RP-expressing cells under hypoxic condition as reported by Gaude et al.^42^, because the percentages of ^13^C_5_-labeled isocitric acid, cis-aconitic acid, and citrate were increased after ^13^C_5_-glutamine labeling. Although malate and succinate will be also generated by reductive carboxylation from glutamine via oxaloacetate, we assume that these TCA intermediates are primarily produced from glutamine using TCA cycle as the basal amount of citrate was substantially reduced by hypoxia. In addition, COX7RP overexpression may also lead to the enhancement of reductive carboxylation in normoxia through glutamine uptake into TCA cycle, because metabolite contents of malic acid and fumaric acid were elevated by COX7RP-overexpressing MCF7 and Ishikawa cells compared with their corresponding vector-cells. Moreover, we showed that COX7RP increases the expression of enzymes within the 2-oxoglutarate dehydrogenase complex (DLD, DLST, and OGDH) in hypoxia, suggesting that COX7RP overexpression elevates succinate and malate levels through the TCA cycle. These results imply that ATP synthesis is enhanced partly through both mitochondrial respiration and glutaminolysis.

Furthermore, the levels of reduced and oxidized glutathione (GSH/GSSG) were elevated in COX7RP-overexpressing MCF7 and Ishikawa cells. Glutathione is a tripeptide synthesized from glutamate, cysteine, and glycine, and acts as a free radical scavenger. Glutathione metabolism could function protectively and pathogenically in cancer biology^43^. Glutathione detoxifies various xenobiotics and carcinogens, and thus the dysregulation of the glutathione pathway can affect cell survival. In cancer, glutathione protects cells by conferring resistance to chemotherapeutic drugs. Thus, enhanced glutathione synthesis may contribute to the elevated growth of COX7RP-overepressing cancer cells. Indeed, a previous study indicated that a suppression of glutathione synthesis decreased tumor initiation and impaired chemotherapy-induced enrichment of breast cancer stem cells^44^. Moreover, we found that expression levels of GSH-synthesizing enzyme *GCLM* and *GSS* were upregulated in COX7RP-MCF7 cells compared with control cells. Supportively, mice lacking the *Gclm* gene, which encodes for a rate-limiting enzyme involved in glutathione synthesis, showed a decrease in breast cancer initiation^45^. It is tempting to speculate that COX7RP may regulate the cancer stem cell-like phenotype by increasing glutathione synthesis under hypoxic conditions.

COX7RP has a key role in the growth of estrogen-dependent breast cancers by activating mitochondrial respiration through facilitating supercomplex assembly. Zhang et al.^46^ reported that COX7RP is expressed in breast cancer cells. They also found by in vitro experiments that COX7RP is induced by cellular stresses, and promotes energy production and breast cancer cell growth. We showed the in vivo role of COX7RP in tumor formation and therapy resistance in breast cancer cells. A recent report revealed that mitochondrial function including biogenesis and ATP production confers a tamoxifen-resistant phenotype to breast cancer cells^47^. We propose that COX7RP promotes mitochondrial respiration during hypoxia and enhances supercomplex assembly, leading to the efficient supply of energy to the cell. Therefore, COX7RP overexpression could allow for the sustainable growth of MCF7 cells during hypoxia. Taken together, we showed that COX7RP could be an important therapeutic target in breast and endometrial cancers.

## Methods

### Cell Culture

MCF7 and 293 T cells were obtained from American Type Culture Collection (Rockville, MD). Ishikawa cells were kindly provided by Dr. Masato Nishida (Kasumigaura Medical Center, Ibaraki, Japan). MCF7, Ishikawa, and 293 T cells were maintained in Dulbecco’s modified Eagle medium (DMEM) supplemented with 10% fetal calf serum (FCS) at 37 °C in 5% CO_2_ and a humidified atmosphere; and were authenticated by short tandem repeat (STR) analysis (BEX, Tokyo, Japan). Clones resistant to tamoxifen (OHTR) were established from MCF7 cells by long-term (>3 months) culture with 1 μm OHT. For stable transfections, MCF7 and Ishikawa cells were transfected with Flag-tagged human COX7RP (Flag-COX7RP) and parental vector pcDNA3 (Invitrogen, San Diego, CA) with only the Flag tag inserted (pcDNA3-Flag). Two clones for each construct and cell line containing either pcDNA3-Flag (vector-MCF7 #1 and #2, and vector-Ishikawa #9 and #10) or Flag-COX7RP (COX7RP-MCF7 #22 and #33, and COX7RP-Ishikawa #1 and #28) were selected by G418. All cells were confirmed to be mycoplasma-free using the e-Myco Mycoplasma PCR Detection Kit (Cosmo Bio, Tokyo, Japan).

### siRNA transfection and western blot analysis

siRNA duplexes targeting *COX7RP*, *OGDH*, *DLST*, *DLD*, *MDH1*, and *MDH2* were synthesized using an algorithm that significantly improves the target specificity of siRNA, in particular, by efficiently estimating off-target sequences^48^. A non-targeting control siRNA (siControl) used in this study was purchased from RNAi Inc. (Tokyo, Japan)^48^. The siRNA sequences were detailed in Supplementary Table 9. siRNAs were transfected into cells using RNAiMAX (Invitrogen, Carlsbad, CA, USA) according to the manufacturer’s instructions. Cell lysates and mitochondrial fractions were prepared in a sample buffer for sodium dodecyl sulfate-polyacrylamide gel electrophoresis (SDS-PAGE), heated at 100 °C for 15 min, and subjected to SDS-PAGE and western blotting analysis using antibodies for COX7RP (1:1000 dilution)^2^, RISP (1:5000 dilution, ab14746, Abcam, Cambridge, MA), COX1 (1:5000 dilution, ab14705, Abcam), COX4 (1:3000 dilution, 4844, Cell signaling, Danvers, MA), and β-actin (1:3000 dilution, AC-74, Sigma-Aldrich, St. Louis, MO).

### Patients and tumor tissue preparation

A total of 168 breast cancer specimens and 59 endometrial cancer specimens were obtained from Japanese female patients at the Department of Surgery, Tohoku University Hospital (Sendai, Japan). Nonpathologic breast tissues (10 specimens) and endometrial tissues (21 specimens) were also available for examination. The ethics committees of Tohoku University School of Medicine approved the research protocols and informed consent was obtained from these patients before surgery in the institution. Total RNA was extracted from these tumors and nonpathologic tissues using an ISOGEN reagent (Nippon Gene, Tokyo, Japan) for quantitative RT-PCR. Evaluation of COX7RP immunohistochemistry was performed on 3-μm sections of the formalin-fixed paraffin-embedded tissue specimens using COX7RP antibody^2,49^. The activity of mitochondrial respiratory chain enzyme complex IV (COX activity) in breast cancer tissues was assessed as follows^50^. Namely, frozen tissue sections were permeabilized with 0.01% saponin and incubated for 15 min in 0.1 m phosphate buffer, pH 7.0 containing 4 nm 3,3’-diaminobenzidine tetra hydrocholoride (DAB) (Sigma-Aldrich), 100 µm reduced cytochrome *c* (Sigma-Aldrich), 2 µg ml^−1^ of catalase (Sigma-Aldrich).

### Quantitative RT-PCR

One microgram of total RNA was reverse-transcribed using poly(dT)20 primer and SuperScript II (Invitrogen). Real-time quantitative RT-PCR (qRT-PCR) for human *COX7RP*, *OGDH*, *DLST*, *DLD*, *MDH1*, *MDH2*, *GCLM*, *GSS*, *GAPDH*, and *36B4* mRNA was performed using a StepOnePlus real-time PCR system (Applied Biosystems, Foster City, CA) using SYBR Green as a fluorescent probe. The primer sequences are detailed in Supplementary Table 10. The reaction mixture for qRT-PCR contained 1% of the reverse-transcribed product together with the SYBR Green PCR master mix (3.5 mm MgCl_2_, 300 μm deoxynucleoside-5’-triphosphate, 0.25 IU hot goldStart enzyme, and SYBR Green) (Applied Biosystems). qRT-PCR was performed for 40 cycles for the indicated genes and cDNA amplification was normalized to *GAPDH* or *36B4*. The results were shown as means ± SD from triplicate experiments.

### Tumor growth in athymic mice

All animal experiments were approved by the Animal Care and Use Committee of Saitama Medical University and conducted in accordance with the Guidelines and Regulations for the Care and Use of Experimental Animals by Saitama Medical University. MCF7, Ishikawa, and OHTR cells (1 × 10^7^ cells per 0.15 ml phosphate-buffered saline) suspended in Matrigel (BD Biosciences, San Jose, CA) were injected subcutaneously into female athymic mice (8-week-old BALB/c nu/nu). Particularly, in order to examine estrogen-dependent growth of MCF7 cells, mice were ovariectomized for 2 weeks prior to the injection of MCF7 cells and administered with E_2_ (1 μg/kg) or vehicle twice a week. We calculated tumor volumes twice a week by measuring the tumor radii. When the tumor volume reached 150 mm^3^, mice were treated with 5 μg siCOX7RP or siControl twice a week. Fifty microliters of a solution containing the siRNAs, 4 μl of GeneSilencer Reagent (Gene Therapy Systems, La Jolla, CA), and DMEM was injected into the tumor. At the end of the experiments, a mitochondria-enriched fraction was prepared from the tumors and analyzed by immunoblotting using an anti-COX7RP antibody (1:1000 dilution)^2^. In experiments using the COX7RP-overexpressing MCF7 and Ishikawa cells, each cell suspension was injected into female athymic mice.

### Cell proliferation

For estrogen-dependent growth, MCF7 and Ishikawa cells were plated at 4 × 10^4^ cells per well in 96-well plates in phenol red-free DMEM containing 1% dextran charcoal-stripped FCS and transfected with 50 nm or 5 nm siRNAs or a negative control (siControl) (RNAi, Tokyo, Japan) for 12 h. Subsequently, 10 nm 17β-estradiol (E_2_) or vehicle was added to the culture medium and cellular growth was assessed after 24, 48, and 72 h. For cell growth under hypoxic conditions, cells were plated at 4 × 10^4^ cells per well in 96-well plates in DMEM. Hypoxic stimulation was produced with ambient oxygen concentrations of 1% (using a controlled incubator with CO_2_/O_2_ monitoring and CO_2_/N_2_ gas sources). For cell growth under pyruvate (10 mm) or cell-permeable diethylsuccinate (2.5 mm) supplementation, cells were also plated at 4 × 10^4^ cells per well in 96-well plates in DMEM. Cell growth was evaluated by measuring cellular DNA content using bisbenzimidazole (Hoechst 33258, Invitrogen) in 96-well plates^51^.

### Cell cycle analysis

Cells were incubated with DMEM under normoxic or hypoxic (1% O_2_) conditions for 24 h before analysis. Trypsinized cells were resuspended in hypotonic propidium iodide solution (50 μg ml^−1^) containing 0.1% sodium citrate and 0.1% Triton X-100, and analyzed on a FACScan flow cytometer (Becton Dickinson, Mountain View, CA).

### COX activity and mitochondrial DNA quantification

For the cytochemical analysis of COX activity, cells were grown on coverslips, fixed in 4% glutaraldehyde, and stained for COX activity with an incubating solution (0.1 m phosphate buffer, pH 7.2, 2 μg ml^−1^ of catalase, 1 mg ml^−1^ of cytochrome *c*, 0.5 mg ml^−1^ of DAB) for 2 h at room temperature. The resulting signal densities were quantitated by the NIH image software package. Relative quantification of mitochondrial DNA (mtDNA) levels was shown as the ratio of the mitochondrial *MTF3212*/*R3319* gene to the nuclear-encoded *B2M* (*β2-microglobulin*). The measurement was performed on the StepOnePlus real-time PCR system by using the primer sets (Supplementary Table 10).

### Measurement of ROS production

Cells seeded in 24-well plates were exposed to hypoxia (1% O_2_) in the presence or absence of 1 mm H_2_O_2_ for 8 h. The mitochondrial ROS probe, MitoSOX (2.5 μm, Molecular Probes, Carlsbad, CA), were then added to the cultures and they further incubated at 37 °C for 20 min. Fluorescence intensity was measured by a fluorometry assay (ARVO X, PerkinElmer, Waltham, MA).

### Metabolite measurements

MCF7 and Ishikawa cells stably transfected with COX7RP or empty vector were cultured in normoxic or hypoxic conditions, washed twice with 5% mannitol solution, and the extracts were isolated with methanol containing Internal Standard Solution (Human Metabolome Technologies, Yamagata, Japan). For tracer experiments, cells were cultured in [U-^13^C]-labeled glutamine for 24 h and the extracts were also isolated. The samples were then passed through a 5-kDa-cutoff filter. The extracted metabolites were concentrated and subjected to CE-TOFMS and triple quadrupole mass spectrometer (QqQ-MS)^52^. Cellular contents of 2-oxoglutaric acid and succinic acid were also determined using the enzymatic kits for α-ketoglutarate (Sigma-Aldrich) and succinate (Roche, Basel, Switzerland), respectively. NAD(H) levels in mitochondria were estimated using a kit for NAD/NADH (Dojindo, Kumamoto, Japan).

### ATP synthesis

Cells were incubated in 150 mm KCl, 25 mm Tris-HCl, pH 7.4, 2 mm EDTA, 10 mm potassium phosphate, 0.1 mm MgCl_2_, and 0.1% bovine serum albumin, with 50 μg ml^−1^ digitonin. Mitochondria were energized using 1 mm malate and 1 mm pyruvate as substrates in the presence of 1 mm ADP and 0.15 mm of an adenylate kinase inhibitor. P1,P5-di(adenosine)pentaphosphate, and incubated for 10 min at 37 °C. Aliquots (50 μl) were resuspended in 25 mm Tris-HCl, pH 7.4, and boiled for 2 min. Parallel incubations were also carried out in the presence of 2 μg ml^−1^ oligomycin to measure mitochondrial-specific ATP synthesis. ATP measurement was performed using a kit (Cell ATP Assay reagent, FUJIFILM, Osaka, Japan)^53^.

### BN-PAGE

Mitochondria were isolated from cells by differential centrifugation and then resuspended in 10 μl of a buffer containing 50 mm Bis-Tris and 1 m 6-aminocaproic acid^2,54^. Digitonin (digitonin/protein ratio of 4 g g^−1^) was added to solubilize the mitochondria. After a 30 min incubation at 4 °C, the samples were centrifuged at 22,000 × *g* and the solubilized proteins were obtained from the supernatant. The solubilized proteins were then supplemented with 1 μl of sample buffer (5% Coomassie Brilliant Blue G-250 in 0.5 m 6-aminocaproic acid). BN-PAGE was performed as described previously^2^ and subsequent immunoblotting was performed according to standard protocols. The blots were probed with anti-NDUFA9 (1:5000 dilution, 459100, Invitrogen), anti-RISP (1:5000 dilution, ab14746, Abcam), anti-COX1 (1:5,000 dilution, ab14705, Abcam), Fp70 (1:5000 dilution, 459200, Invitrogen), and anti-COX7RP antibodies (1:3000 dilution)^2^. Signal intensities for mitochondrial respiratory complexes and supercomplexes were quantified by normalization with their corresponding Fp70 using triplicated western blot analysis.

### Measurements of oxygen consumption

Cells were seeded (5 × 10^3^ cells/well) in 96-well plates (Seahorse Biosciences, Billerica, MA) and cultured in normoxia (20% O_2_) or hypoxia (1%, 5%, or 10% O_2_) overnight before measuring their OCR and ECAR. For knockdown experiments, cells were also transfected with 10 nm siRNAs for 48 h. 1 h before the assay, culture medium was replaced with XF base medium containing the substrates (1 mm sodium pyruvate, 2 mm glutamate, and 25 mm glucose (Seahorse Biosciences). The rate of oxygen consumption (OCR) was measured at 37 °C on an XF Extracellular Flux Analyzer (Seahorse Bioscience) using the Cell Mito Stress Test Kit (Seahorse Bioscience). The baseline (basal) OCR was measured three times before and three times after each sequential injection of oligomycin (2 μm), carbonyl cyanide 4-(trifluoromethoxy)phenylhydrazone (0.25 μm), and rotenone and antimycin A (both 0.5 μm). ECAR was analyzed before the addition of oligomycin. The complex II-meditated respiratory activity was measured as follows. Cells were incubated in MAS-bovine serum albumin (BSA) solution (70 mm sucrose, 220 mm mannitol, 10 mm KH_2_PO_4_, 5 mm MgCl_2_, 2 mm HEPES, 1 mm EGTA, and 4 mg ml^−1^ BSA) and co-injected with 0.01% saponin, 1 mm ADP, 1 μm rotenone, and 10 mm succinate^55^. The OCR levels were monitored after the injection.

### Microarray analysis

Gene expression in the COX7RP-MCF7 and vector-MCF7 cells was measured with the Affymetrix GeneChip (HuGene-1_0-st-v1) according to the manufacturer’s protocol. The microarray data have been deposited in Gene Expression Omnibus under accession number GSE124524. The DAVID Functional Annotation Clustering Tool (http://david.abcc.ncifcrf.gov/summary.jsp) was used to perform a global analysis of gene expression and to sort differentially expressed genes into pathways and clusters of functionally related genes.

### Statistics

We determined significant differences between two groups by unpaired two-sided Student’s *t* test and by Mann–Whitney *U* test for nonparametric samples. For comparisons among stable cell lines, we performed a two-way analysis of variance. Survival analyses were carried out using the Kaplan–Meier method and log-rank test. We considered a value of *P* < 0.05 to be statistically significant.

### Reporting summary

Further information on research design is available in the Nature Research Reporting Summary linked to this article.

## Supplementary information

Supplementary Information

Description of Additional Supplementary Files

Supplementary Data 1

Supplementary Data 2

Reporting Summary

Source Data

## Data Availability

The accession number for microarray data presented in Supplementary Table 8 is GSE124524. Data of metabolomic analysis and tracer experiment performed in this study are provided by Supplementary Data 1 and 2, respectively. The source data underlying Figs. 2–8, Supplementary Tables 3–7, and Supplementary Figs. 2–14 and 16–21 are provided as a Source Data file. All the other data supporting the findings of this study are available from the corresponding authors upon reasonable request.
