# Supplementary material for: Mitochondrial supercomplex assembly promotes breast and endometrial tumorigenesis by metabolic alterations and enhanced hypoxia tolerance
Source: Nat Commun. 2019 Sep 11;10:4108. doi: 10.1038/s41467-019-12124-6 (PMC6739376; doi:10.1038/s41467-019-12124-6)
Supplement: Supplementary file 1 — Supplementary Information [file 41467_2019_12124_MOESM1_ESM.pdf]

**Supplementary Information**

# Mitochondrial supercomplex assembly promotes breast and endometrial tumorigenesis by metabolic alterations and enhanced hypoxia tolerance

Ikeda *et al.*

**Supplementary Data 1.** Metabolomic profiles of COX7RP transformants of MCF7 and Ishikawa cells in normoxia or hypoxia. Steady-state levels of 116 metabolites were analyzed in COX7RP-MCF7 #22, vector-MCF7 #1, COX7RP-Ishikawa #28, and vector-Ishikawa #9 cells by capillary electrophoresis time-of-flight mass spectrometry (CE-TOFMS).

**Supplementary Data 2.** Tracer experiment with [U-$^{13}$C]-labeled glutamine in COX7RP-MCF7 #22 and vector-MCF7 #1 cells in normoxia or hypoxia. Cells were cultured in [U-$^{13}$C]-labeled glutamine for 24 h and the extracts were analyzed by CE-TOFMS.

**Supplementary Table 1.** Association between COX7RP immunoreactivity and clinicopathological parameters in 168 breast carcinomas.

| | COX7RP immunoreactivity | | *P* |
|---|---|---|---|
| | + (*n* = 71) | − (*n* = 97) | |
| Age* (years) | 54.9 ± 11.3 | 54.5 ± 11.8 | 0.84 |
| Menopausal status | | | |
| Premenopausal | 45 | 62 | |
| Postmenopausal | 26 | 35 | 0.94 |
| Stage | | | |
| I | 12 | 29 | |
| II | 37 | 50 | |
| III | 22 | 18 | 0.06 |
| Tumor size* (cm) | 3.7 ± 3.3 | 3.3 ± 3.4 | 0.43 |
| Lymph node status | | | |
| Positive | 40 | 32 | |
| Negative | 31 | 65 | **0.003** |
| Histological grade | | | |
| 1 (well) | 17 | 20 | |
| 2 (moderate) | 32 | 42 | |
| 3 (poor) | 22 | 35 | 0.76 |
| ERα status | | | |
| Positive | 58 | 61 | |
| Negative | 13 | 36 | **0.01** |
| ERα LI* (%) | 56.4 ± 35.0 | 36.8 ± 33.9 | **0.0004** |
| PR LI* (%) | 36.0 ± 33.0 | 27.9 ± 29.5 | 0.10 |
| HER2 status | | | |
| Positive | 18 | 21 | |
| Negative | 53 | 76 | 0.57 |
| Ki-67 LI* (%) | 21.0 ± 15.0 | 19.6 ± 17.3 | 0.60 |

*; Data are presented as means ± s.e.m. All other values represent the number of cases.
*P*-values (Chi-square test) less than 0.05 were considered as significant, and are in boldface. ER; estrogen receptor. PR; progesterone receptor. LI; labeling index.

**Supplementary Table 2.** Univariate and multivariate analyses of breast cancer-specific survival in 168 breast cancer patients examined.

| | Univariate | Multivariate | |
|---|---|---|---|
| Variable | *P* | *P* | Relative risk (95% CI) |
| Lymph node status (positive / negative) | **< 0.0001** | **0.001** | 4.1 (1.9 - 9.1) |
| HER2 status (positive / negative) | **0.0003** | 0.11 | |
| Histological grade (3 / 1, 2) | **0.001** | **0.01** | 2.5 (1.2 - 5.2) |
| COX7RP immunoreactivity (positive / negative) | **0.002** | **0.001** | 4.1 (1.8 - 9.3) |
| ERα status (negative / positive) | **0.02** | **0.048** | 2.2 (1.0 - 4.8) |
| Tumor size (≧2.0 cm / <2.0 cm) | 0.07 | | |
| Ki-67 LI (≧10 / <10) | 0.10 | | |

Statistical analysis was evaluated by a proportional hazard model (Cox). Data considered significant ($P < 0.05$) in the univariate analysis are in boldface, and further evaluated by the multivariate analysis. CI; confidence interval. ER; estrogen receptor.

**Supplementary Table 3.** Signal intensities for mitochondrial respiratory complexes and supercomplexes in MCF-7 transfectants.

| Cells/culture condition | $CI+CIII_2+CIV_n$[a] | $CI+CIII_2$[a] | $CIII_2+CIV_n$[b] | $CIII_2$[c] | CIV[d] |
|---|---|---|---|---|---|
| Vector-MCF7 #1/ normoxia | 0.27 ± 0.06 | 0.17 ± 0.03 | 0.10 ± 0.01 | 0.40 ± 0.06 | 0.79 ± 0.05 |
| Vector-MCF7 #1/ hypoxia | 0.15 ± 0.03 | 0.14 ± 0.04 | 0.09 ± 0.01 | 0.39 ± 0.14 | 0.77 ± 0.28 |
| COX7RP-MCF7 #22/ normoxia | 0.64 ± 0.07†† | 0.23 ± 0.03 | 0.14 ± 0.01†† | 0.53 ± 0.07 | 0.94 ± 0.09 |
| COX7RP-MCF7 #22/ hypoxia | 0.55 ± 0.09** | 0.29 ± 0.04* | 0.14 ± 0.01** | 0.60 ± 0.03 | 1.08 ± 0.12 |

[a] Signal intensities for mitochondrial supercomplexes $CI+CIII_2+CIV_n$ and $CI+CIII_2$ detected by NDUFA9 antibody were quantified by normalization with the corresponding Fp70 signal using triplicated western blot analysis.
[b] Signal intensities for mitochondrial supercomplex $CIII_2+CIV_n$ detected by RISP antibody were quantified by normalization with the corresponding Fp70 signal using triplicated western blot analysis.
[c] Signal intensities for mitochondrial complex $CIII_2$ detected by RISP antibody were quantified by normalization with the corresponding Fp70 signal using triplicated western blot analysis.
[d] Signal intensities for mitochondrial complex CIV detected by COX1 antibody were quantified by normalization with the corresponding Fp70 signal using triplicated western blot analysis.
Data are presented as means ± SD ($n$ = 3 biologically independent samples).
††, $P < 0.01$ compared with Vector-MCF7 #1/normoxia (Student's $t$-test).
*, $P < 0.05$; **, $P < 0.01$ compared with Vector-MCF7 #1/hypoxia (Student's $t$-test).
Source data are provided as a Source Data file.

**Supplementary Table 4.** COX7RP signal intensities at the positions of mitochondrial respiratory complexes and supercomplexes in MCF-7 transfectants.

| Cells/culture condition | $CI+CIII_2+CIV_n$ | $CI+CIII_2$ | $CIII_2+CIV_n$ | $CIII_2$ or $CIV_n$ | CIV |
|---|---|---|---|---|---|
| Vector-MCF7 #1/ normoxia | 0.19 ± 0.03 | 0.10 ± 0.01 | 0.10 ± 0.01 | 0.18± 0.05 | 0.19 ± 0.04 |
| Vector-MCF7 #1/ hypoxia | 0.14 ± 0.03 | 0.14 ± 0.01 | 0.09 ± 0.01 | 0.26 ± 0.03 | 0.29 ± 0.14 |
| COX7RP-MCF7 #22/ normoxia | 0.28 ± 0.04† | 0.12 ± 0.01 | 0.14 ± 0.01† | 0.27 ± 0.07 | 0.20 ± 0.01 |
| COX7RP-MCF7 #22/ hypoxia | 0.25 ± 0.01** | 0.15 ± 0.02 | 0.11 ± 0.01* | 0.32 ± 0.04 | 0.24 ± 0.08 |

Signal intensities of COX7RP at the positions for $CI+CIII_2+CIV_n$, $CI+CIII_2$, $CIII_2+CIV_n$, $CIII_2$ or $CIV_n$, and CIV were quantified by normalization with the corresponding Fp70 signal using triplicated western blot analysis. Data are presented as means ± SD ($n$ = 3 biologically independent samples).

†$P < 0.05$ compared with Vector-MCF7 #1/normoxia (Student's *t*-test).

*$P < 0.05$; **$P < 0.01$ compared with Vector-MCF7 #1/hypoxia (Student's *t*-test).

Source data are provided as a Source Data file.

**Supplementary Table 5.** Signal intensities for mitochondrial respiratory complexes and supercomplexes in OHTR cells.

| Cells/culture condition | $CI+CIII_2+CIV_n$ | $CI+CIII_2$ | $CIII_2+CIV_n$ | $CIII_2$ |
|---|---|---|---|---|
| MCF7/vehicle | 0.70 ± 0.06 | 0.39 ± 0.10 | 0.11 ± 0.02 | 0.47 ± 0.17 |
| MCF7/OHT | 0.43 ± 0.14 | 0.38 ± 0.03 | 0.05 ± 0.02 | 0.44 ± 0.13 |
| OHTR/vehicle | 0.98 ± 0.11† | 0.57 ± 0.06 | 0.28 ± 0.02†† | 0.54 ± 0.05 |
| OHTR/OHT | 0.95 ± 0.12** | 0.51 ± 0.11 | 0.31 ± 0.03** | 0.70 ± 0.22 |

Signal intensities at the positions for $CI+CIII_2+CIV_n$, $CI+CIII_2$, $CIII_2+CIV_n$, and $CIII_2$, all of which were detected by RISP antibody, were quantified by normalization with the corresponding Fp70 signals using triplicated western blot analysis. Data are presented as means ± SD ($n$ = 3 biologically independent samples).

†$P < 0.05$; ††$P < 0.01$ compared with Vector-MCF7 #1/normoxia (Student's *t*-test).

**$P$ <0.01, compared with Vector-MCF7 #1/hypoxia (Student's *t*-test).

Source data are provided as a Source Data file.

**Supplementary Table 6.** Signal intensities for mitochondrial respiratory complexes and supercomplexes in Ishikawa transfectants.

| Cells/culture condition | $CI+CIII_2+CIV_n$[a] | $CI+CIII_2$[a] | $CIII_2+CIV_n$[b] | $CIII_2$[c] |
|---|---|---|---|---|
| Vector-Ishikawa #9/ normoxia | 39.91 ± 11.32 | 18.93 ± 2.29 | 6.20 ± 0.28 | 13.06 ± 1.92 |
| Vector-Ishikawa #9/ hypoxia | 16.24 ± 2.75 | 15.21 ± 3.00 | 4.95 ± 0.30 | 14.68 ± 4.15 |
| COX7RP-Ishikawa #28/ normoxia | 34.58 ± 10.96 | 18.04 ± 4.63 | 7.22 ± 0.95 | 19.76 ± 3.87 |
| COX7RP-Ishikawa #28/ hypoxia | 34.55 ± 10.73* | 18.92 ± 2.65 | 6.67 ± 0.94* | 19.23 ± 0.91 |

[a] Signal intensities for mitochondrial supercomplexes $CI+CIII_2+CIV_n$ and $CI+CIII_2$ detected by NDUFA9 antibody were quantified by normalization with the corresponding Fp70 signal using triplicated western blot analysis.
[b] Signal intensities for mitochondrial supercomplex $CIII_2+CIV_n$ detected by RISP antibody were quantified by normalization with the corresponding Fp70 signal using triplicated western blot analysis.
[c] Signal intensities for mitochondrial complex $CIII_2$ detected by RISP antibody were quantified by normalization with the corresponding Fp70 signal using triplicated western blot analysis.
Data are presented as means ± SD ($n$ = 3 biologically independent samples).
*, $P < 0.05$ compared with Vector-MCF7 #1/hypoxia (Student's $t$-test).
Source data are provided as a Source Data file.

**Supplementary Table 7.** COX7RP signal intensities at the positions of mitochondrial respiratory complexes and supercomplexes in Ishikawa transfectants.

| Cells/culture condition | $CI+CIII_2+CIV_n$ | $CI+CIII_2$ | $CIII_2+CIV_n$ | $CIII_2$ or $CIV_n$ |
|---|---|---|---|---|
| Vector-MCF7 #1/ normoxia | 14.77 ± 0.38 | 14.27 ± 0.89 | 19.41 ± 3.86 | 16.19 ± 3.82 |
| Vector-MCF7 #1/ hypoxia | 10.01 ± 2.00 | 11.00 ± 1.87 | 11.67 ± 2.78 | 15.03 ± 1.08 |
| COX7RP-MCF7 #22/ normoxia | 20.76 ± 3.05 | 16.45 ± 1.06 | 28.17 ± 2.20 | 25.05 ± 7.31 |
| COX7RP-MCF7 #22/ hypoxia | 20.63 ± 3.79* | 16.21 ± 2.94 | 28.50 ± 6.78* | 20.03 ± 3.22 |

Signal intensities of COX7RP at the positions for $CI+CIII_2+CIV_n$, $CI+CIII_2$, $CIII_2+CIV_n$, $CIII_2$ or $CIV_n$, and CIV were quantified by normalization with the corresponding Fp70 signal using triplicated western blot analysis. Data are presented as means ± SD ($n$ = 3 biologically independent samples).

*$P < 0.05$ compared with Vector-MCF7 #1/hypoxia (Student's $t$-test).

Source data are provided as a Source Data file.

**Supplementary Table 8. KEGG pathway analysis of up- and down-regulated genes in COX7RP-MCF7 cells during hypoxia[a)].**

**(a) Upregulated KEGG pathway genes in hypoxic COX7RP-MCF7 cells compared with Vector-MCF7 cells**

| Term | Count | *P* value | Genes |
|---|---|---|---|
| Cell cycle | 6 | 3.41E-03 | CDC6, BUB1, TTK, SMAD2, CCNA2, WEE1 |
| Vibrio cholerae infection | 4 | 9.31E-03 | ATP6V1C1, SLC12A2, ATP6V0A4, ATP6V1D |
| Metabolic pathways | 18 | 1.75E-02 | MGAT4A, NADK2, ODC1, ST6GAL1, MAT2A, PIK3C2A, POLE, ACSS2, LPIN1, ATP6V1D, MMAB, ATP6V1C1, SQLE, DLD, FASN, LCLAT1, ATP6V0A4, ACSL3 |
| Collecting duct acid secretion | 3 | 2.06E-02 | ATP6V1C1, ATP6V0A4, ATP6V1D |
| Glycerophospholipid metabolism | 4 | 4.32E-02 | GPD2, LCLAT1, LPIN1, LPCAT3 |

**(b) Downregulated KEGG pathway genes in hypoxic COX7RP-MCF7 cells compared with Vector-MCF7 cells**

| Term | Count | *P* value | Genes |
|---|---|---|---|
| Legionellosis | 5 | 6.83E-04 | TNF, IL18, PYCARD, HSPA6, HSPA1A |
| Influenza A | 7 | 1.82E-03 | ICAM1, TNF, IL18, JUN, PYCARD, HSPA6, HSPA1A |
| Systemic lupus erythematosus | 5 | 1.79E-02 | TNF, HIST1H2BD, HIST3H2A, HIST1H4D, HIST1H3F |
| Antigen processing and presentation | 4 | 1.92E-02 | TNF, HSPA6, HSPA1A, KIR2DL2 |
| African trypanosomiasis | 3 | 2.53E-02 | ICAM1, TNF, IL18 |
| Rheumatoid arthritis | 4 | 2.82E-02 | ICAM1, TNF, IL18, JUN |
| MAPK signaling pathway | 6 | 4.18E-02 | TNF, JUN, HSPA6, HSPA1A, GADD45B, GADD45A |
| Malaria | 3 | 5.22E-02 | ICAM1, TNF, IL18 |
| NOD-like receptor signaling pathway | 3 | 6.41E-02 | TNF, IL18, PYCARD |
| Inflammatory bowel disease (IBD) | 3 | 8.34E-02 | TNF, IL18, JUN |
| p53 signaling pathway | 3 | 9.02E-02 | PMAIP1, GADD45B, GADD45A |

[a)]RNAs were prepared from COX7RP-MCF7 #22 and vector-MCF7 #1 cells cultured under normoxic or hypoxic (1% $O_2$) conditions for 24 h and subjected to microarray analysis using HuGene-1_0-st-v1 (Affymetrics). KEGG pathway analysis was performed using the DAVID Functional Annotation Clustering Tool (http://david.abcc.ncifcrf.gov/summary.jsp). *P* values were calculated using Fisher's exact test.

**Supplementary Table 9.** Sequences of siRNAs used.

| siRNA | Sense sequence | Antisense sequence |
|---|---|---|
| siCOX7RP #1 | 5'-GUGGGAGGGACCAUCUACUGC-3' | 5'-AGUAGAUGGUCCCUCCCACAG-3' |
| siCOX7RP #2 | 5'-GCUGAACACAGGCUUGUUAAU-3' | 5'-UAACAAGCCUGUGUUCAGCCU-3' |
| siOGDH #1 | 5'-CGGAAGCCGUUAAUUAUCUUC-3' | 5'-AGAUAAUUAACGGCUUCCGGA-3' |
| siOGDH #2 | 5'-GGGCUCUAGCGGAGUACAUGG-3' | 5'-AUGUACUCCGCUAGAGCCCAG-3' |
| siDLST #1 | 5'-GUGAUUGACGACACAACCAAA-3' | 5'-UGGUUGUGUCGUCAAUCACUG-3' |
| siDLST #2 | 5'-GAUUGACGACACAACCAAAGA-3' | 5'-UUUGGUUGUGUCGUCAAUCAC-3' |
| siDLD #1 | 5'-GUCCGAAGUUCGCUUGAAUUU-3' | 5'-AUUCAAGCGAACUUCGGACAU-3' |
| siDLD #2 | 5'-GACGACCCUUUACUAAGAAUU-3' | 5'-UUCUUAGUAAAGGGUCGUCGG-3' |
| siMDH1 #1 | 5'-CUGUCAUCAAGGCUCGAAAAC-3' | 5'-UUUCGAGCCUUGAUGACAGCA-3' |
| siMDH1 #2 | 5'-CGACUCAGUAUCCAGAUGUCA-3' | 5'-ACAUCUGGAUACUGAGUCGAG-3' |
| siMDH2 #1 | 5'-CGCCUGACCCUCUAUGAUAUC-3' | 5'-UAUCAUAGAGGGUCAGGCGGC-3' |
| siMDH2 #2 | 5'-GAGUCAACGUCCCUGUCAUUG-3' | 5'-AUGACAGGGACGUUGACUCGA-3' |
| siControl | 5′-GUACCGCACGUCAUUCGUAUC-3′ | 5′-UACGAAUGACGUGCGGUACGU-3′ |

**Supplementary Table 10.** Sequences of primers used.

| Gene | Forward | Reverse |
|---|---|---|
| COX7RP | 5'-GCCTGCCTGACCAAATGC-3' | 5'-GGGCGATCAGGCAGT-3' |
| OGDH | 5'-GGACGTGGTTGTCGATTTGG-3' | 5'-TGCGTGAACATGGGCTCAT-3' |
| DLST | 5'-GCGGCCCATGATGTACGT-3' | 5'-CACAGCCTCTCTGCCATCAAT-3' |
| DLD | 5'-GTCCAATGCTGGCTCACAAA-3' | 5'-CCAGCCATTCCTTCAACACA-3' |
| MDH1 | 5'-TGGAACCCCAGAGGGAGAGT-3' | 5'-CACCATAGGAGTTGCCATCAGA-3' |
| MDH2 | 5'-CCCATCACAGCAGAAGTTTTCA-3' | 5'-TCACGCCGAAGATTTTGTTG-3' |
| GCLM | 5’-ACGCACAGCGAGGAGCTT-3’ | 5’-GATTTGGGAACTCCATTCATTCA-3’ |
| GSS | 5’-GGGCCTCCTACATCCTCATG-3’ | 5’-CCGTAGCAGGCAATTCTCAAA-3’ |
| GAPDH | 5'-GGTGGTCTCCTCTGACTTCAACA-3' | 5'-GTGGTCGTTGAGGGCAATG-3' |
| 36B4 | 5'-CCACGCTGCTGAACATGCT-3' | 5'-GATGCTGCCATTGTCGAACA-3' |
| MTF3212/R3319 | 5’-CACCCAAGAACAGGGTTTGT-3’ | 5’-TGGCCATGGGTATGTTGTTA-3’ |
| B2M | 5’-TGCTGTCTCCATGTTTGATGTATCT-3’ | 5’-TCTCTGCTCCCCACCTCTAAGT-3’ |

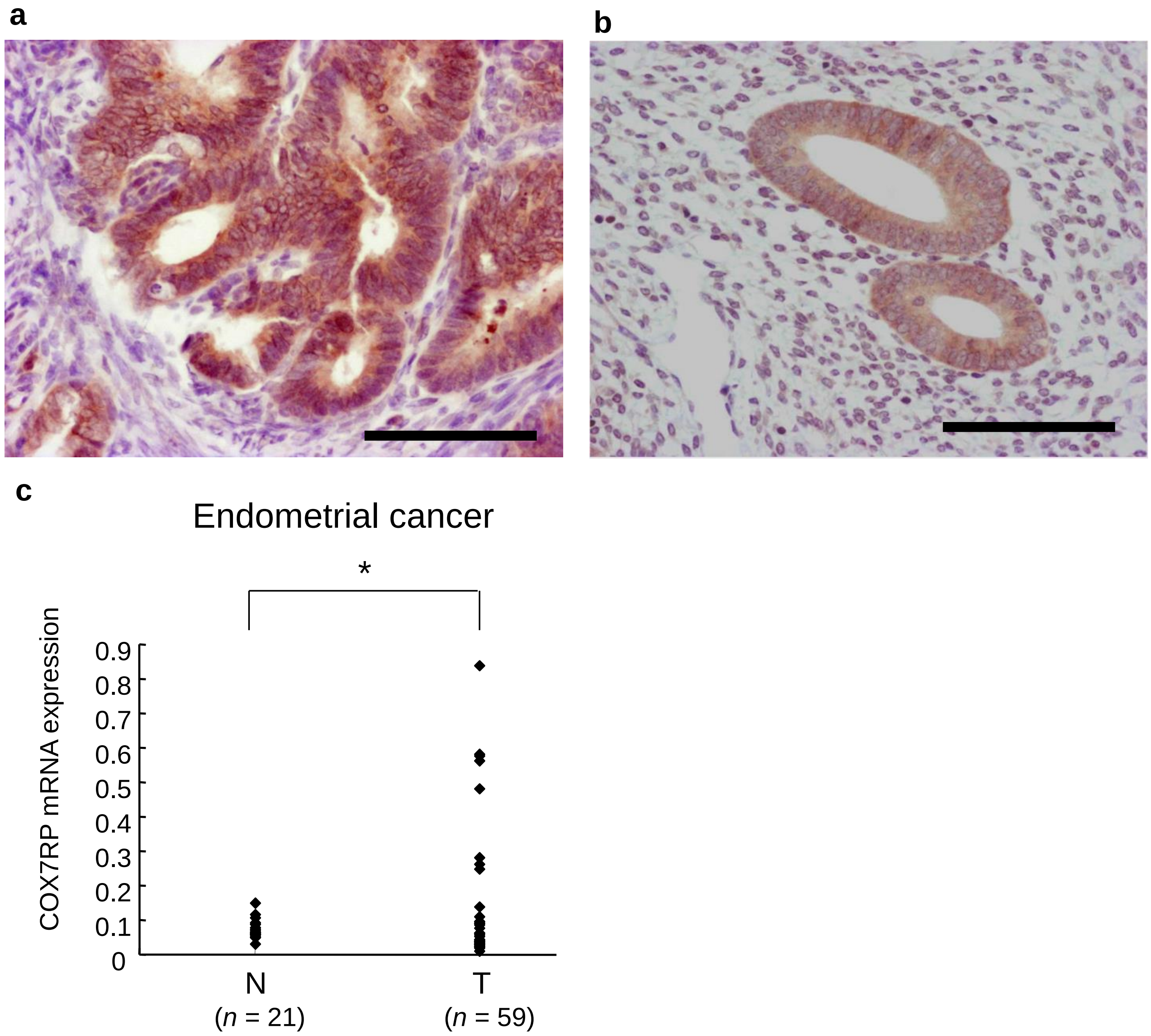


**Supplementary Figure 1 | Increased expression of COX7RP in endometrial cancer.** Sections of human endometrial cancer tissue (**a**) and normal endometrium (**b**) were stained for COX7RP. Scale bar, 100 μm. (**c**) Real-time RT–PCR analysis of *COX7RP* mRNA expression in tumor (T) and normal (N) samples. *$P < 0.05$, Student's *t*-test.

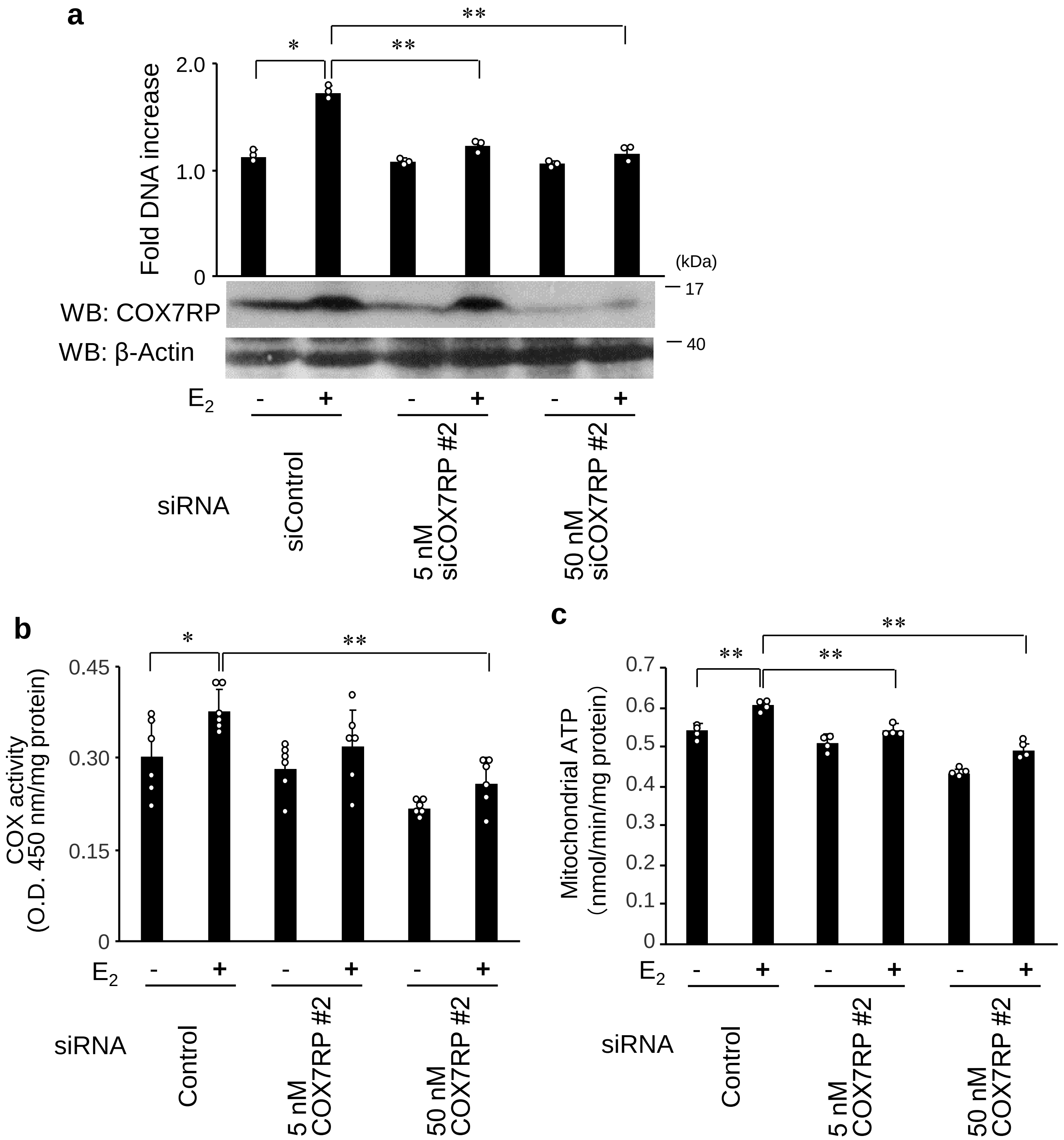


**Supplementary Figure 2 | Inhibition of COX7RP expression by siCOX7RP #2 suppresses estrogen-induced cell proliferation and ATP production in MCF7 cells.** (**a**) siCOX7RP #2 attenuates estrogen-induced COX7RP expression and hormone-dependent growth of MCF7 cells. MCF7 cells were transfected with siCOX7RP #2 or siControl and cell growth was estimated by DNA amount after 72 h ($n$ = 3). Western blot analysis was performed using anti-COX7RP and β-actin antibodies. (**b**) Silencing of COX7RP decreases COX activity. COX7RP activity was assessed in MCF7 cells treated with siCOX7RP #2 or siControl ($n$ = 6). (**c**) Silencing of COX7RP decreases ATP production. ATP production in mitochondria was assessed in MCF7 cells treated with siCOX7RP #2 or siControl ($n$ = 3 independent experiments). Data are presented as means ± SD. *$P$ < 0.05, **$P$ < 0.01, Student's $t$-test. Source data are provided as a Source Data file.

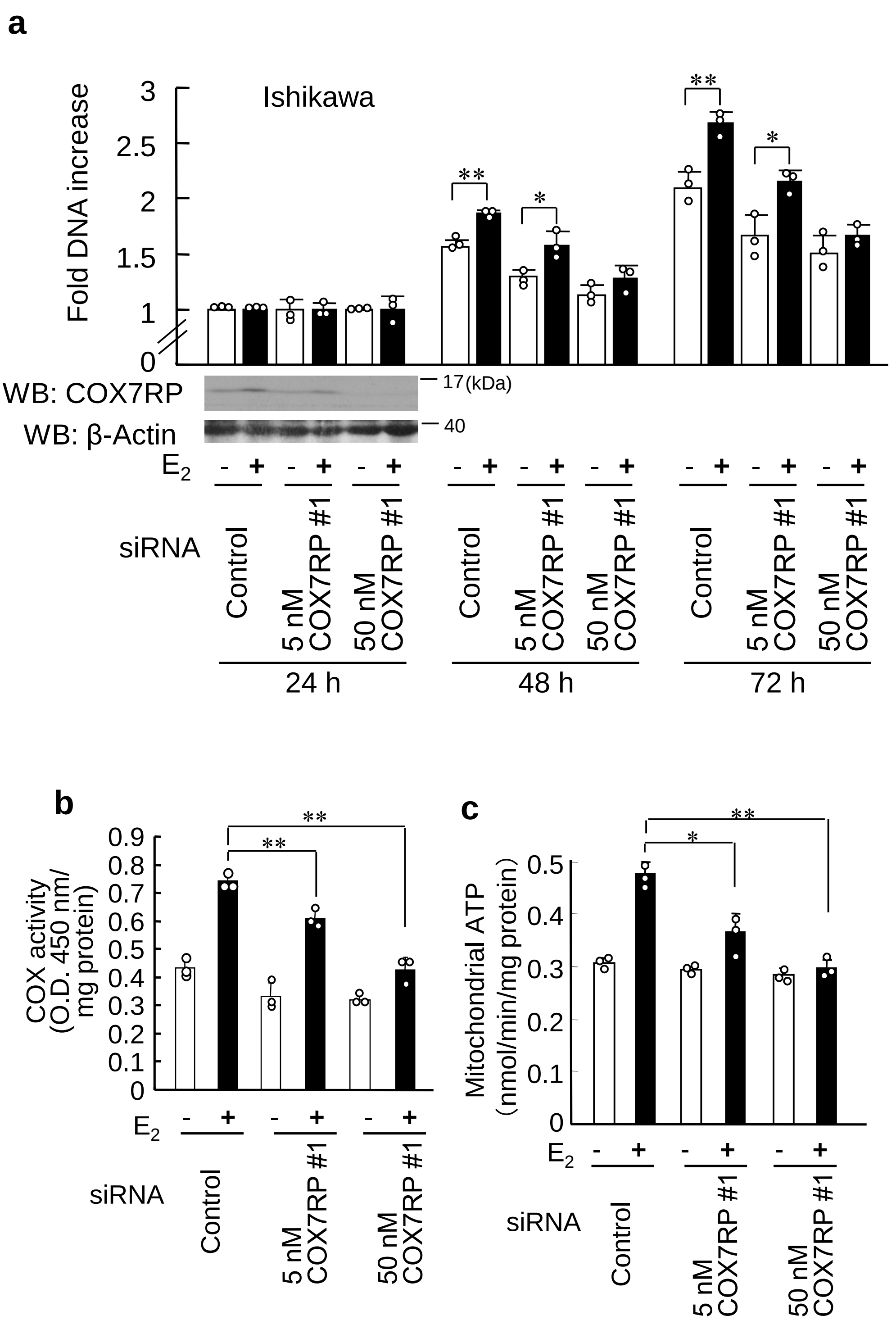


**Supplementary Figure 3 | Inhibition of COX7RP expression suppresses estrogen-induced cell proliferation and ATP production in Ishikawa cells.** (**a**) siCOX7RP attenuates estrogen-induced COX7RP expression and hormone-dependent growth of Ishikawa cells. Ishikawa cells were transfected with siCOX7RP #1 or siControl for the indicated times and cell growth was estimated by DNA amount. Western blot analysis was performed using an anti-COX7RP and β-actin antibodies. (**b**) Silencing of COX7RP decreases COX activity. COX7RP activity was assessed in Ishikawa cells treated with siCOX7RP #1 or siControl. (**c**) Silencing of COX7RP decreases ATP production. ATP production in mitochondria was assessed in Ishikawa cells treated with siCOX7RP #1 or siControl. Data are presented as means ± SD ($n$ = 3 independent experiments). *$P < 0.05$, **$P < 0.01$, Student's $t$-test. Source data are provided as a Source Data file.

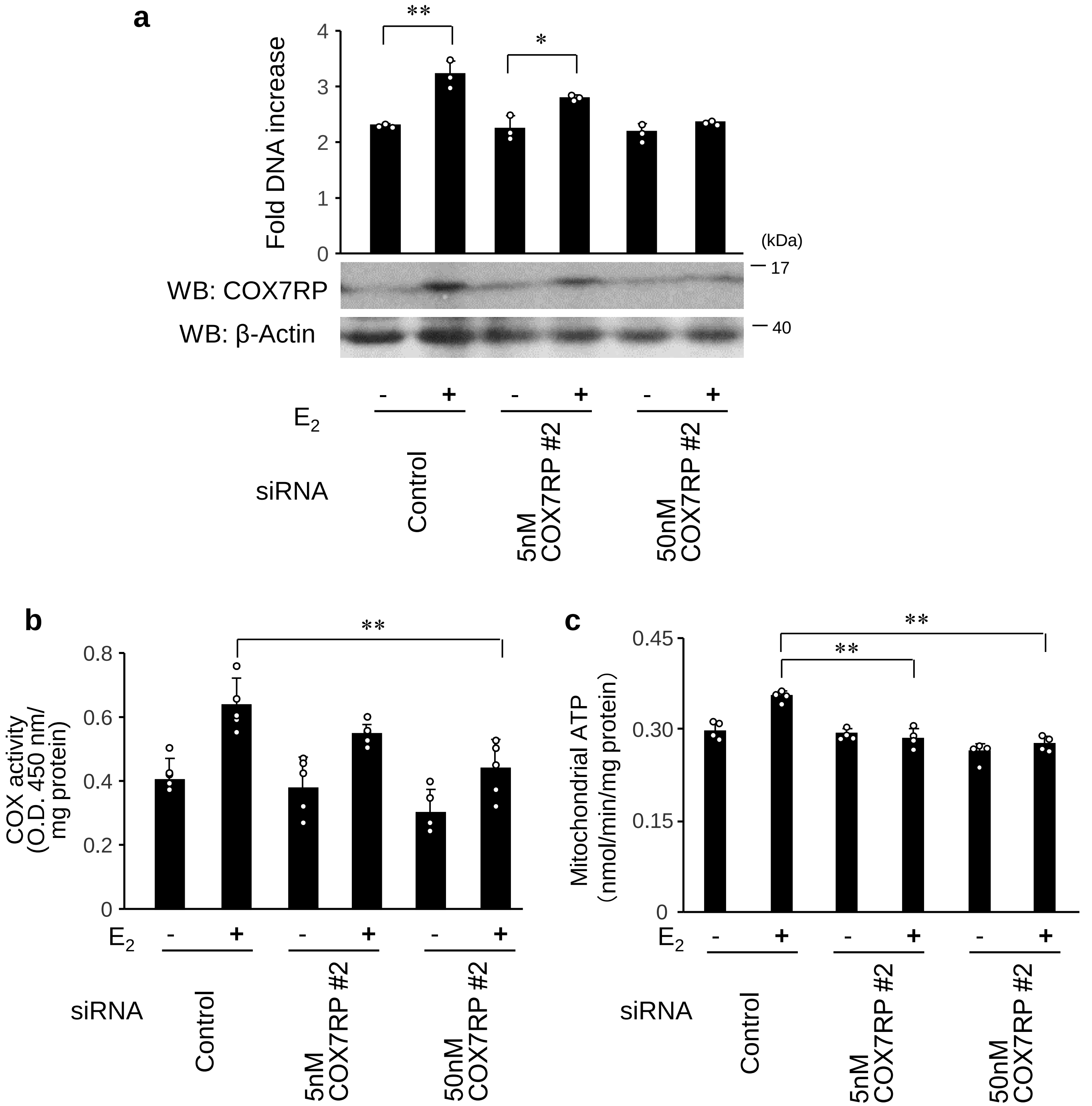


**Supplementary Figure 4 | Inhibition of COX7RP expression by siCOX7RP #2 suppresses estrogen-induced cell proliferation and ATP production in Ishikawa cells.** (**a**) siCOX7RP #2 attenuates estrogen-induced COX7RP expression and hormone-dependent growth of Ishikawa cells. Ishikawa cells were transfected with siCOX7RP #2 or siControl and cell growth was estimated by DNA amount after 72 h ($n$ = 3 independent experiments). Western blot analysis was performed using an anti-COX7RP and β-actin antibodies. (**b**) Silencing of COX7RP decreases COX activity. COX7RP activity was assessed in Ishikawa cells treated with siCOX7RP #2 or siControl ($n$ = 5 independent experiments). The COX activity was measured by cytochemistry. (**c**) Silencing of COX7RP decreases ATP production. ATP production in mitochondria was assessed in Ishikawa cells treated with siCOX7RP #2 or siControl. Data are presented as means ± SD ($n$ = 4 independent experiments). *$P$ < 0.05, **$P$ < 0.01, Student's $t$-test. Source data are provided as a Source Data file.

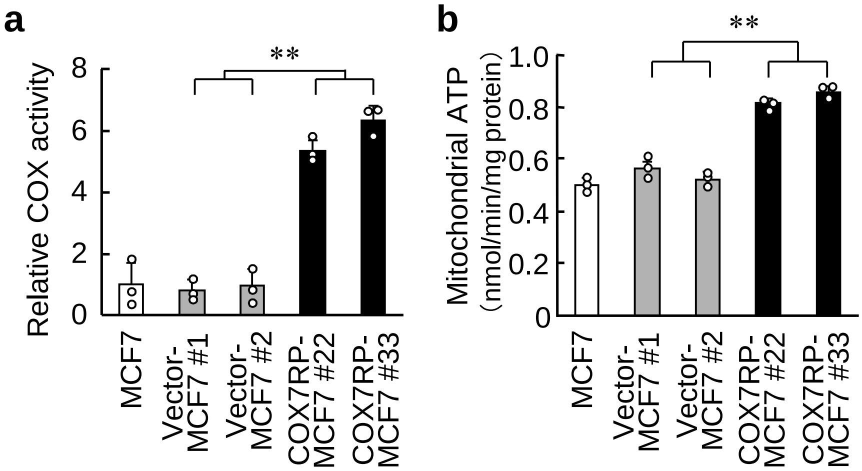


**Supplementary Figure 5 | COX7RP stimulates COX activity and ATP production in MCF7 cells.** (**a**) Increased COX activity of COX7RP-MCF7 cells. The COX activity was measured by cytochemistry. (**b**) Increased ATP production rate in mitochondria of COX7RP-MCF7 cells. Data are presented as means ± SD ($n$ = 3 independent experiments). **$P$ < 0.01, Two-way analysis of variance. Source data are provided as a Source Data file.

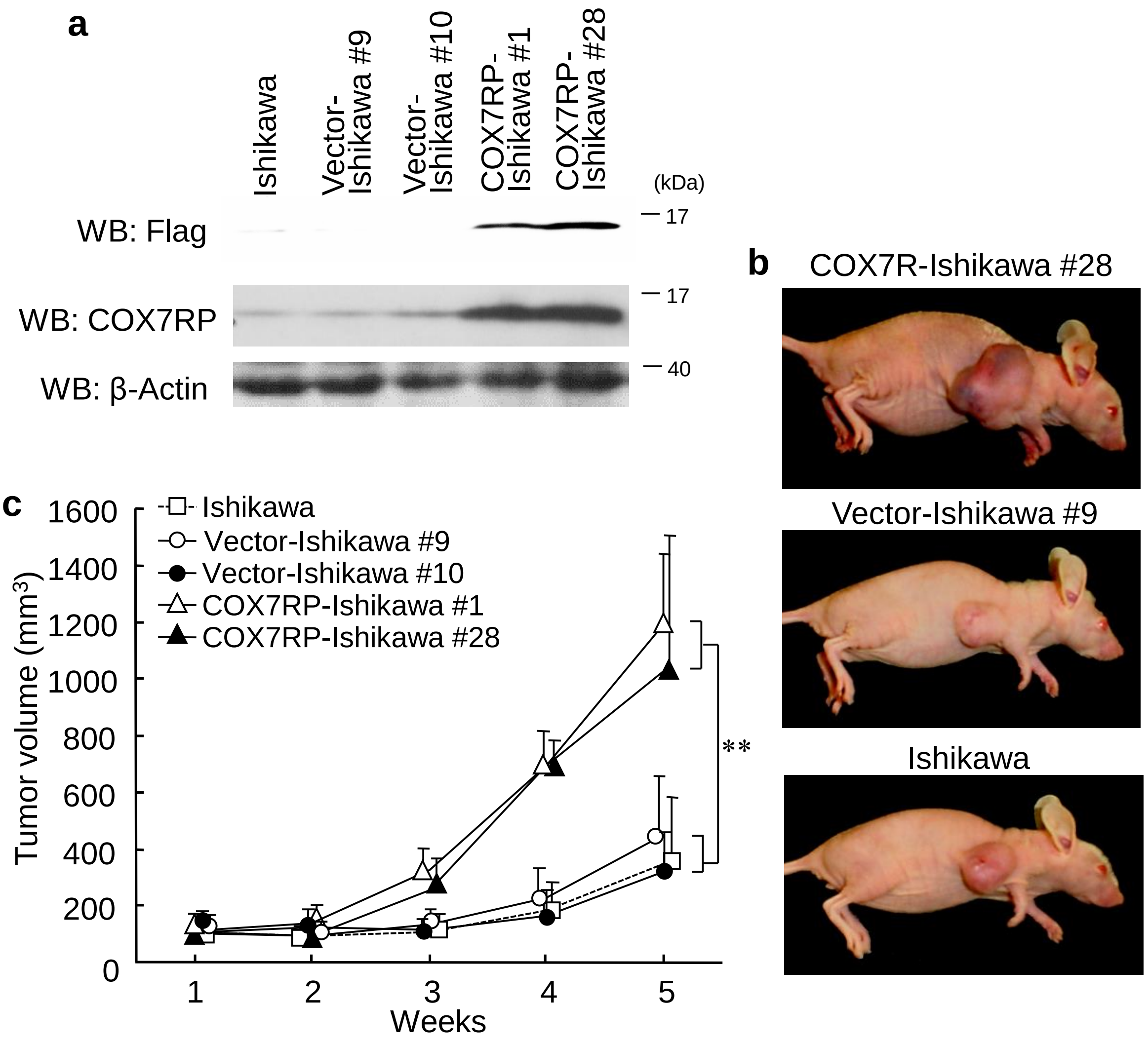


**Supplementary Figure 6 | COX7RP promotes tumor growth of Ishikawa cells *in vivo*.** (**a**) Generation of Ishikawa cells stably expressing COX7RP (COX7RP-Ishikawa #1 and #28) or control vector (vector-Ishikawa #9 and #10). Western blot analysis was performed with anti-Flag, anti-COX7RP, and β-actin antibodies. (**b, c**) Increased tumor growth was observed in athymic mice bearing COX7RP-Ishikawa cells. Athymic mice were inoculated with Ishikawa-derived cells and observed for 5 weeks and the tumor volumes ($mm^3$) were calculated. Data are presented as means ± SD ($n$ = 9 animals). *, $P < 0.05$; **, $P < 0.01$ for COX7RP-Ishikawa cells versus vector-Ishikawa cells (Two-way analysis of variance). Source data are provided as a Source Data file.

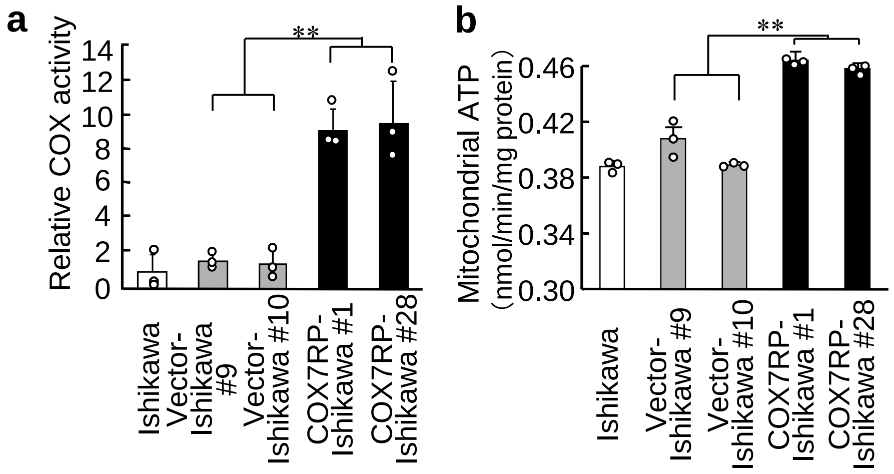


**Supplementary Figure 7 | COX7RP stimulates COX activity and ATP production in Ishikawa cells.** (**a**) Increased-COX activity of COX7RP-Ishikawa cells. The COX activity was measured by cytochemistry. (**b**) Increased ATP production rate in mitochondria of COX7RP-Ishikawa cells. Data are presented as means ± SD ($n$ = 3 independent experiments). **$P < 0.01$, Two-way analysis of variance. Source data are provided as a Source Data file.

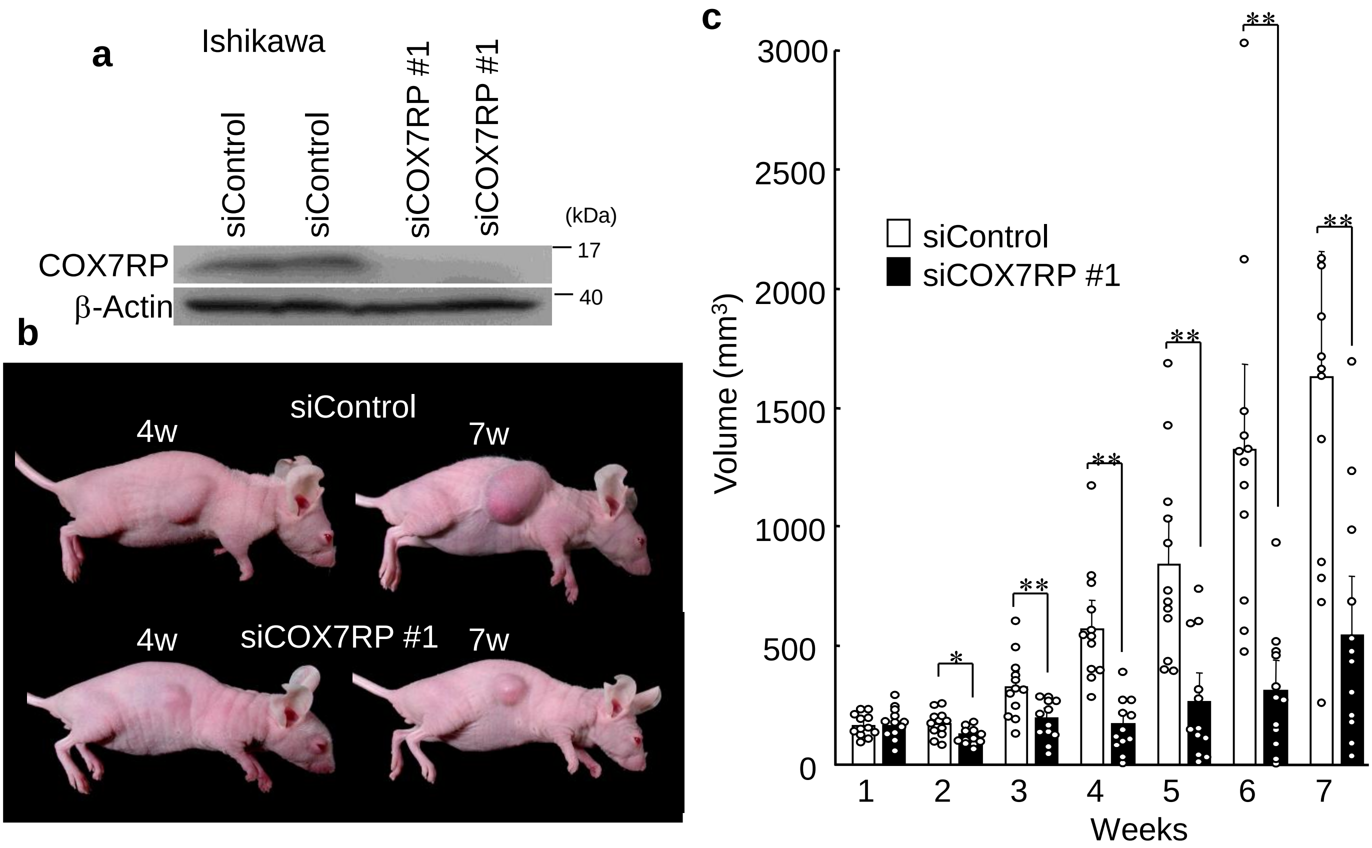


**Supplementary Figure 8 | Inhibition of COX7RP expression suppresses tumor growth induced by Ishikawa cells.** Athymic female mice were inoculated with 2 x $10^7$ Ishikawa cells and then administrated with control siRNA (siControl) or COX7RP siRNA (siCOX7RP #1) (5 mg twice a week). (**a**) COX7RP expression was assessed by western blot analysis using an anti-COX7RP antibody and anti-β-actin antibody as a control in tumors obtained from mice inoculated with Ishikawa cells. (**b**) Reduced tumor size in mice 4 and 7 weeks after inoculation with siControl or siCOX7RP #1. (**c**) Volume ($mm^3$) of tumors caused by Ishikawa cells in nude mice. Data are presented as means ± SEM ($n$ = 12 animals). *$P$ < 0.05; **$P$ < 0.01, Student's $t$-test. Source data are provided as a Source Data file.

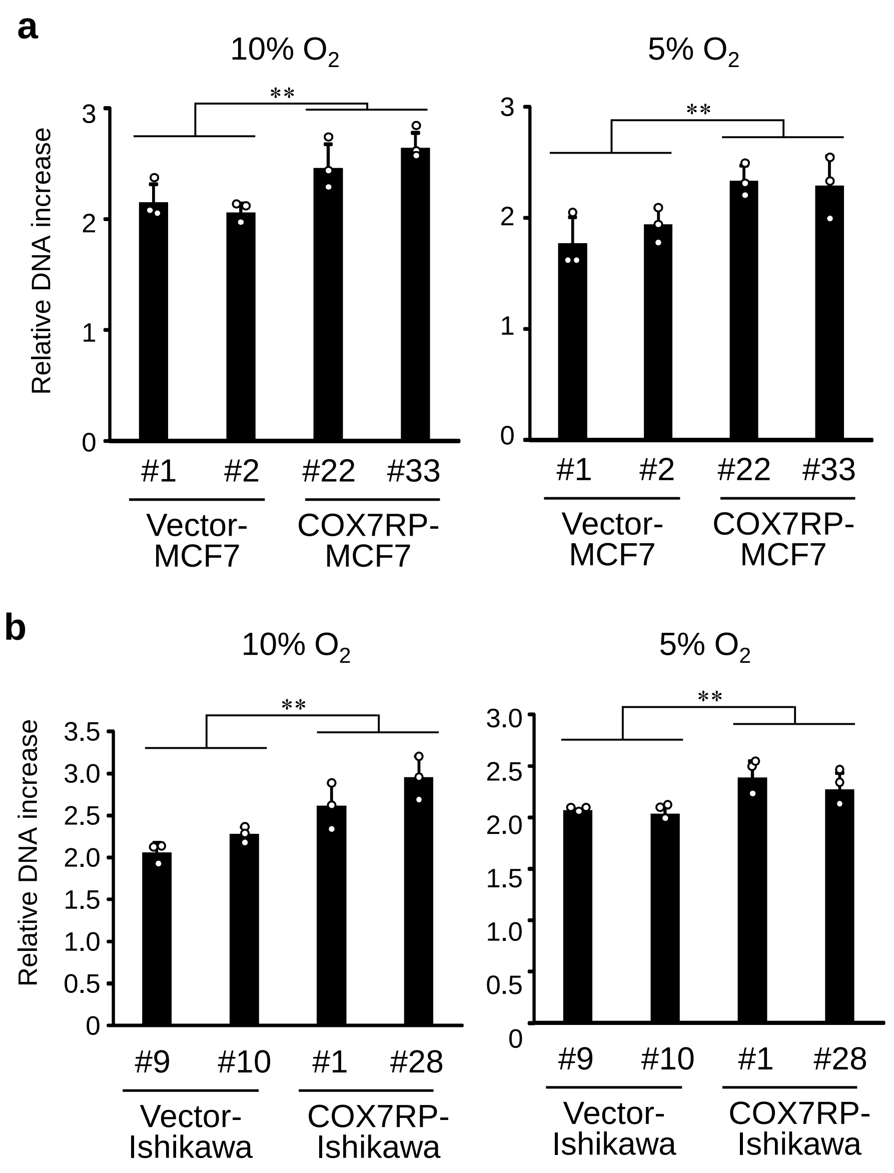


**Supplementary Figure 9 | Effects of oxygen concentration on proliferation of MCF7 and Ishikawa cells stably expressing COX7RP or control vector.** (**a**, **b**) Cell growth of MCF7 (a) and Ishikawa (b) stable transfectants was estimated in reduced oxygen (10% and 5% $O_2$) culture conditions by DNA assay. Data are presented as means ± SD (*n* = 3 independent experiments). ***P* < 0.01, Two-way analysis of variance. Source data are provided as a Source Data file.

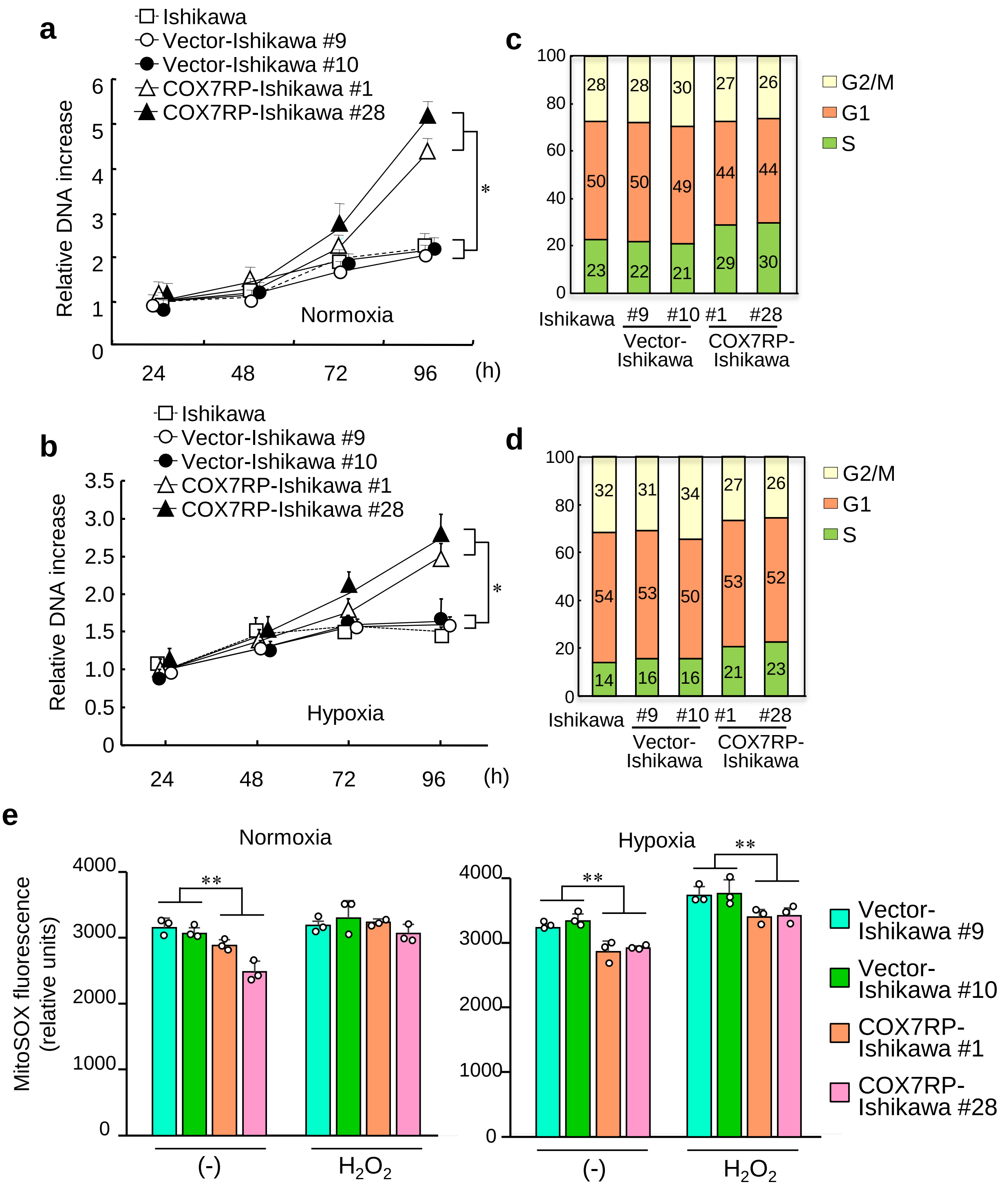


**Supplementary Figure 10 | Overexpression of COX7RP induces hypoxia tolerance and decreases mitochondrial ROS in Ishikawa cells.** (**a**) Growth of COX7RP-Ishikawa cells was promoted under normoxic culture conditions (20% $O_2$). (**b**) Continual growth of COX7RP-Ishikawa cells in hypoxic culture conditions (1% $O_2$). COX7RP overexpression increases the percentage of Ishikawa cells in the proliferation stage of the cell cycle during normoxia (**c**) and hypoxia (**d**). (**e**) Decrease in mitochondrial ROS levels in COX7RP-Ishikawa cells. Cells were exposed to hypoxia in the presence or absence of $H_2O_2$ for 8 h, and then incubated with the mitochondrial ROS probe, MitoSOX. Fluorescence intensity was measured by a fluorimetry assay. Data are presented as means ± SD (*n* = 3 independent experiments). *, *P* < 0.05; **, *P* < 0.01, Two-way analysis of variance. Source data are provided as a Source Data file.

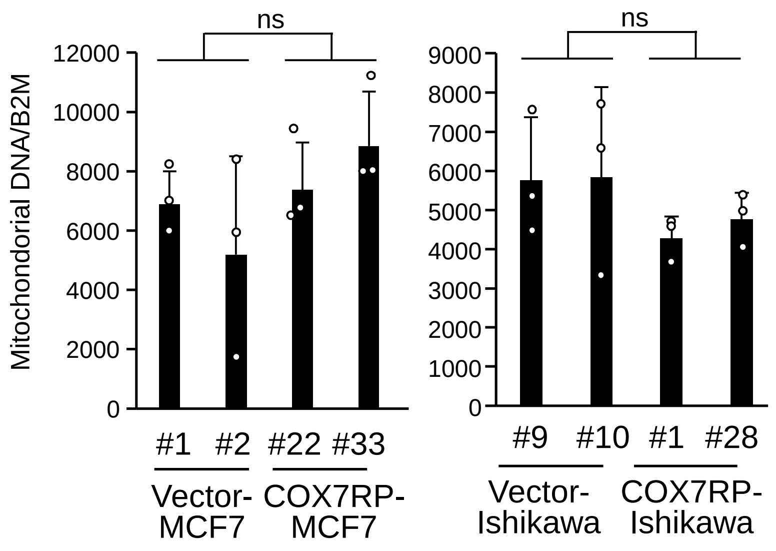


**Supplementary Figure 11 | Mitochondrial DNA amounts in MCF7 and Ishikawa cells stably expressing COX7RP or control vector.** The mitochondrial DNA amount was determined by quantitative PCR using primers for the mitochondrial *MTF3212/R3319* gene and nuclear-encoded *B2M* (*β2-microglobulin*) gene. Relative mitochondrial DNA levels normalized with *B2M* were calculated and data are presented as means ± SD (*n* = 3 independent experiments). ns, not significant (Two-way analysis of variance). Source data are provided as a Source Data file.

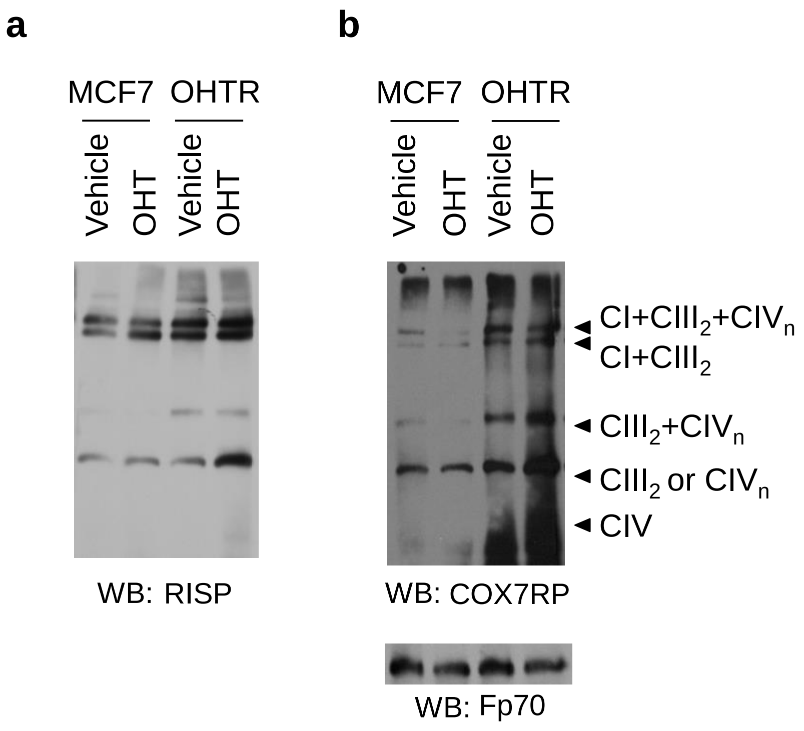


**Supplementary Figure 12 | Mitochondrial respiratory supercomplex was increased in 4-hydroxytamoxifen-resistant breast cancer cells.** (**a**, **b**) Mitochondrial proteins prepared from MCF7 and MCF7-derived tamoxifen-resistant (OHTR) cells treated with 4-hydroxytamoxifen (OHT) or vehicle were solubilized with digitonin and subjected to BN-PAGE. Western blot analysis was performed with antibodies for RISP of complex III, COX7RP of complex IV, and Fp70 of complex II as a loading control. Positions corresponding to the $CI+CIII_2+CIV_n$, $CI+CIII_2$, $CIII_2+CIV_n$, $CIII_2$ or $CIV_n$, and CIV are indicated. Source data are provided as a Source Data file.

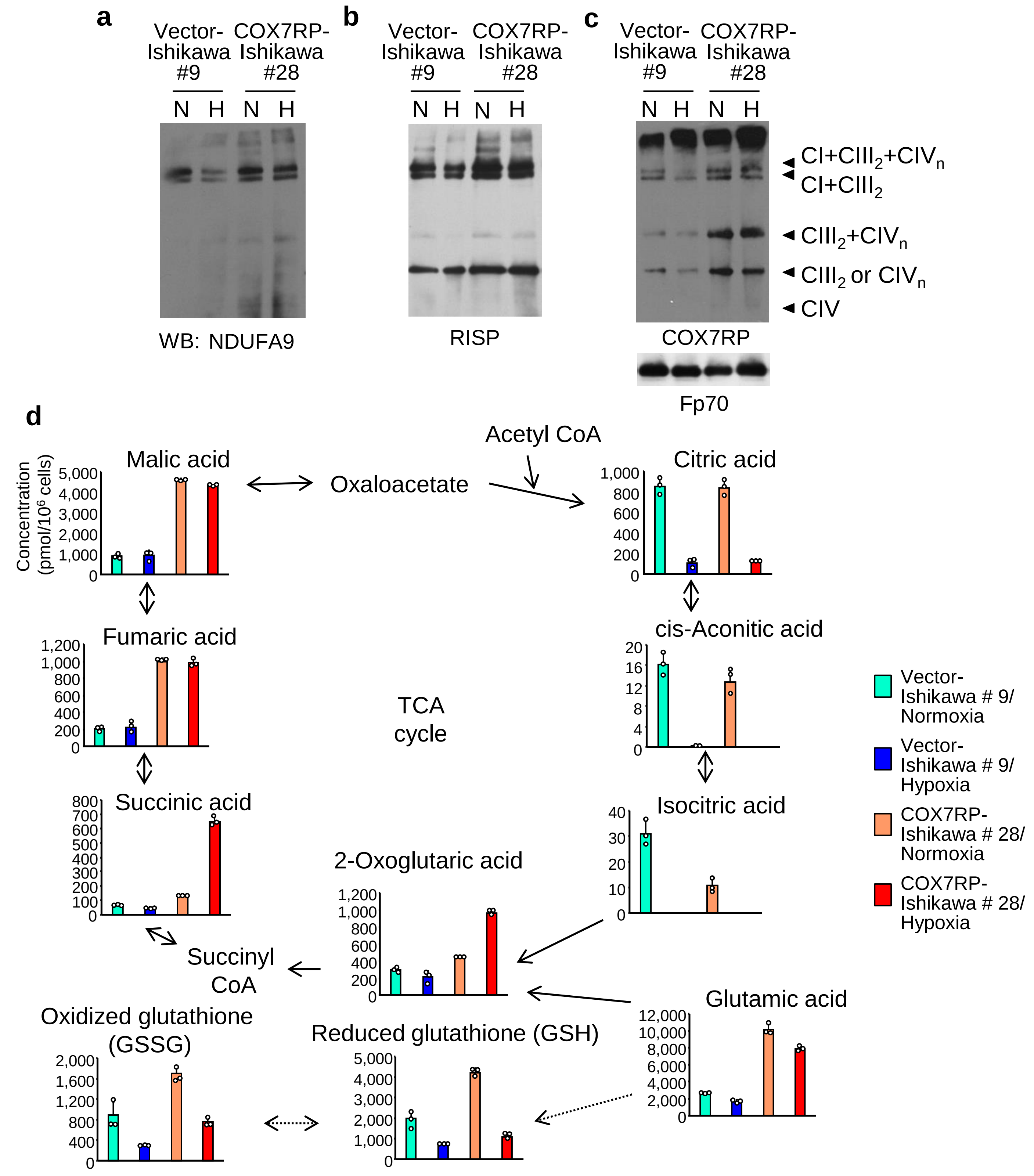


**Supplementary Figure 13 | COX7RP facilitates supercomplex assembly in hypoxia and modulates metabolic pathways in Ishikawa cells.** Mitochondrial proteins of COX7RP-Ishikawa cells treated during normoxia (N) and hypoxia (H: 1% $O_2$) were solubilized with digitonin and subjected to BN-PAGE. Western blot analysis was performed with antibodies for NDUFA9 (**a**), RISP (**b**), and COX7RP or Fp70 (**c**). Positions corresponding to the CI+$CIII_2$+$CIV_n$, CI+$CIII_2$, $CIII_2$+$CIV_n$, $CIII_2$ or $CIV_n$, and CIV are indicated. (**d**) COX7RP-Ishikawa cells were cultured under normoxic or hypoxic conditions for 24 h and cell extracts were subjected to metabolome analysis using capillary electrophoresis time-of-flight mass spectrometry (CE-TOFMS) for cation analysis and capillary electrophoresis-tandem mass spectrometry (CE-MS/MS) for anion analysis. Metabolites in the TCA cycle are schematically shown. Intracellular concentration (pmol/million cells) of key metabolites involved in the TCA cycle and glutathione synthesis pathway are shown as means ± SD ($n$ = 3 biologically independent samples). Source data are provided as a Source Data file.

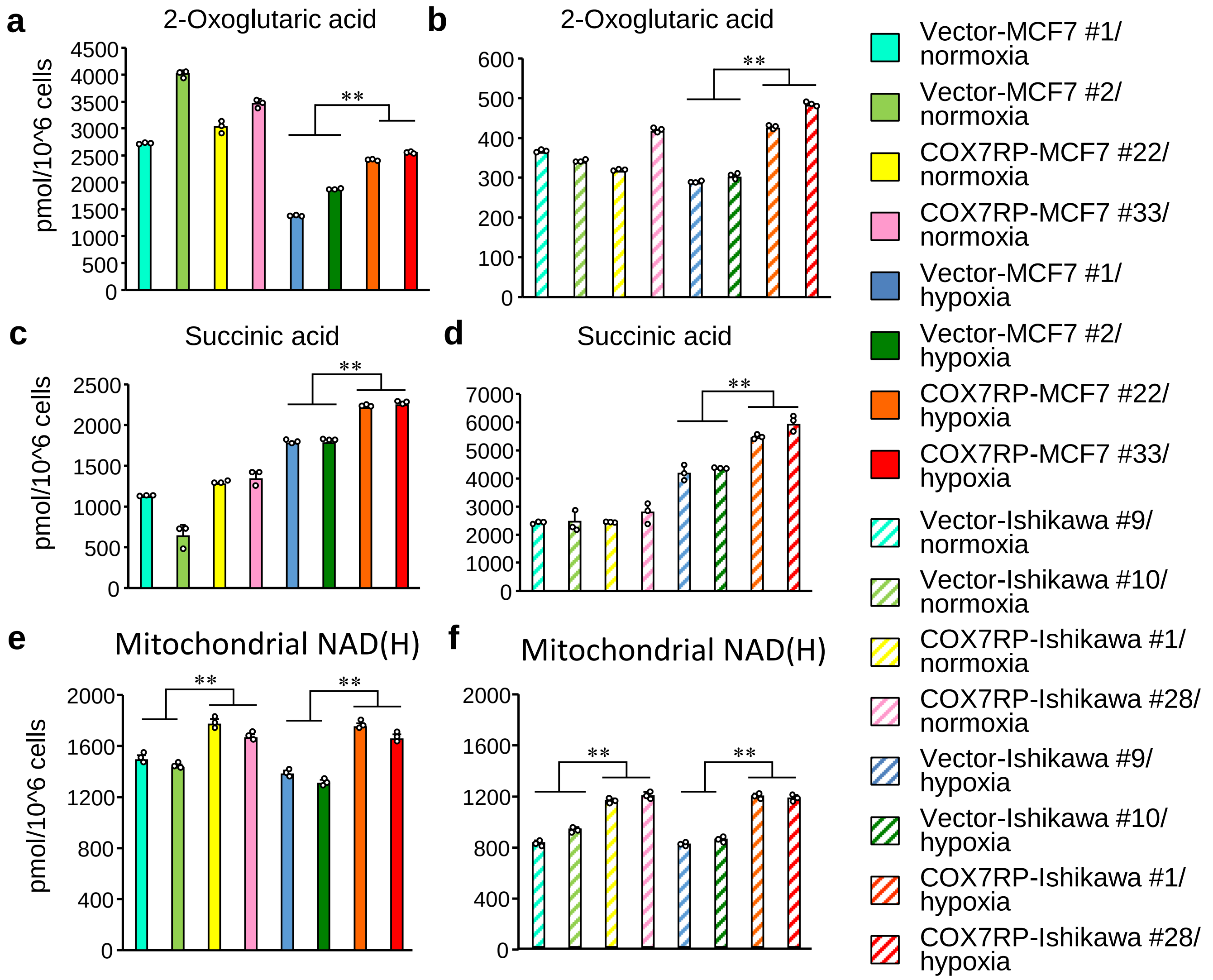


**Supplementary Figure 14 | Amounts of TCA cycle intermediates and mitochondrial NAD(H) in MCF7 and Ishikawa cells stably expressing COX7RP or control vector.** (**a**-**d**) MCF7 and Ishikawa cells stably expressing COX7RP and control vector were cultured under normoxic (20% $O_2$) and hypoxic (1% $O_2$) condition for 24 h. Whole cell lysates were subjected to quantification of 2-oxoglutaric acid (a, b) and succinic acid (c, d) in MCF7 (a, c) and Ishikawa (b, d) transfectants, respectively. (**e**, **f**) Mitochondrial NAD(H) levels in MCF7 and Ishikawa transfectants. Mitochondrial fractions were prepared from the MCF7 (e) and Ishikawa (f) transfectants cultured under normoxic or hypoxic conditions for 24 h and subjected to quantification of NAD(H). Data are presented as means ± SD (*n* = 3 independent experiments). **, *P* < 0.01, Two-way analysis of variance. Source data are provided as a Source Data file.

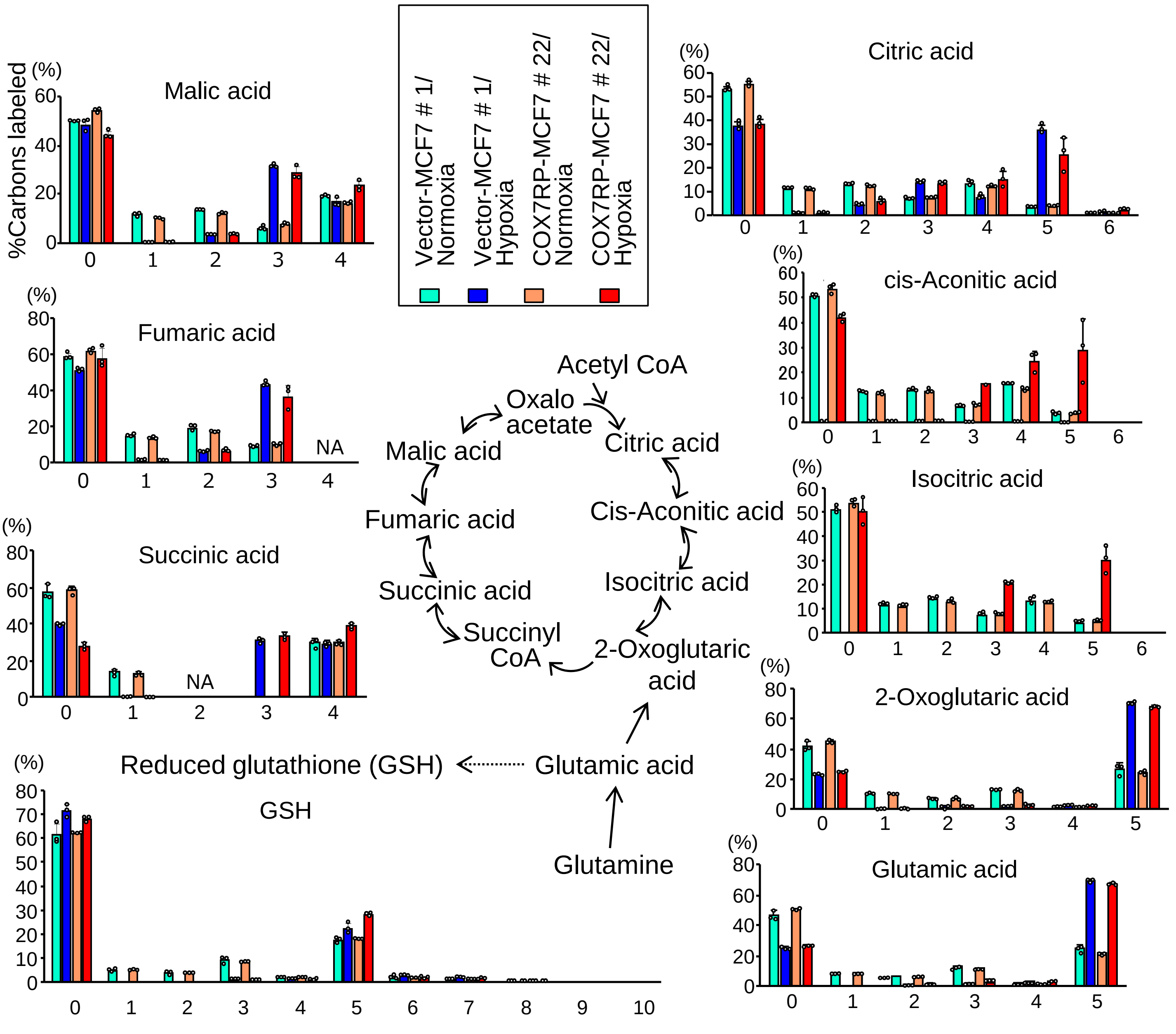


**Supplementary Figure 15 | Relative abundances of $^{13}$C-labeled isotopologues in TCA cycle intermediates in MCF7 stable transfectants 24 h after uptake of $^{13}C_5$-labeled glutamine.** Column charts show relative isotopologue abundances for selected metabolites. The x-axis indicates the number of $^{13}$C-labeled carbons in each isotopologue. Isotopologues for fumaric acid (+ 4) and succinic acid (+ 2) are indistinguishable due to the similar mass number and thus shown as NA (not applicable). Data are presented as means ± SD ($n$ = 3 biologically independent samples. Source data are provided as a Source Data file.

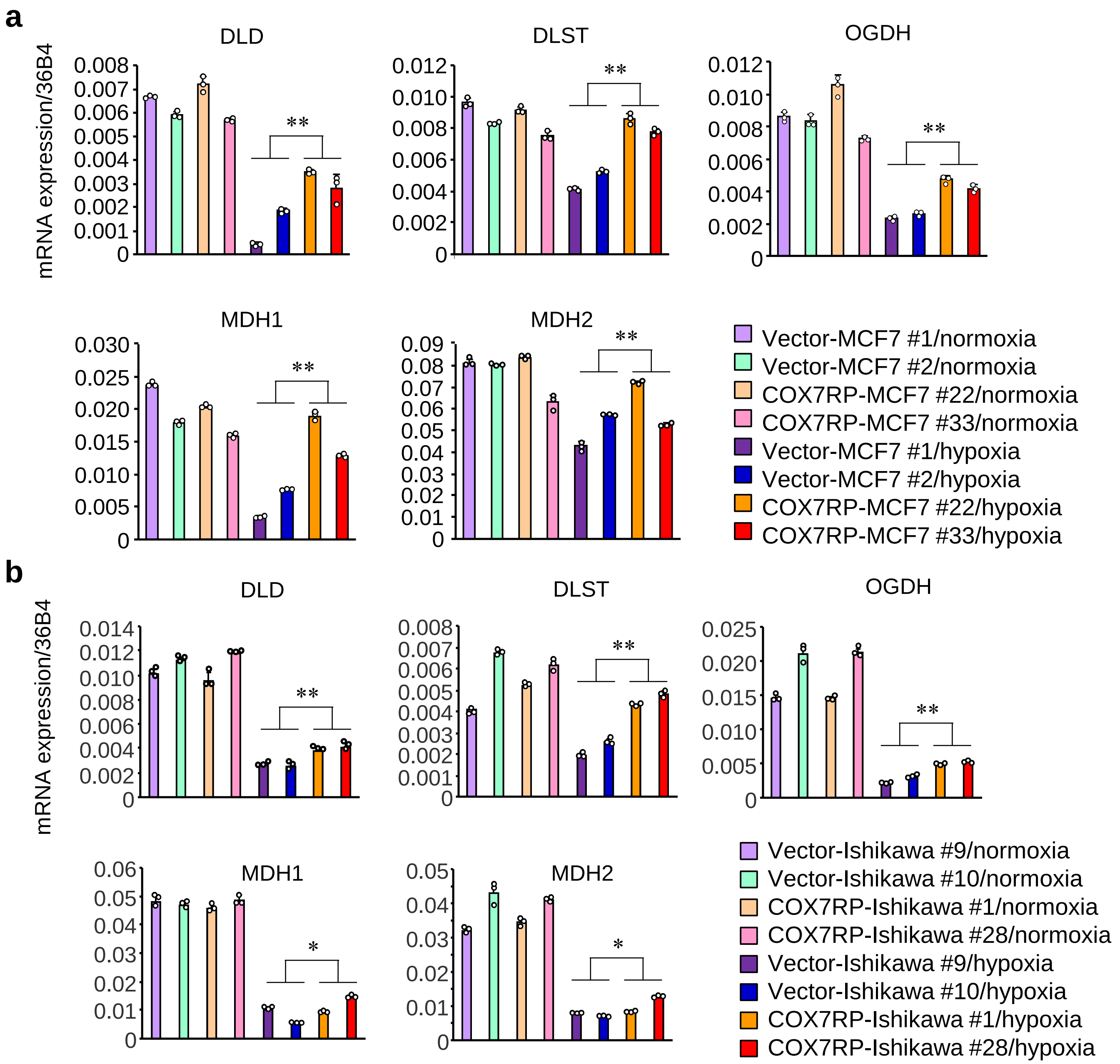


**Supplementary Figure 16 | COX7RP regulates metabolic pathway genes.** RNAs were prepared from COX7RP-MCF7 cells (**a**) and COX7RP-Ishikawa cells (**b**) cultured under normoxic or hypoxic (1% $O_2$) conditions for 24 h and gene expression levels were quantified by qRT-PCR. Data are presented as means ± SD (*n* = 3 independent experiments). **, $P < 0.01$, Two-way analysis of variance. Source data are provided as a Source Data file.

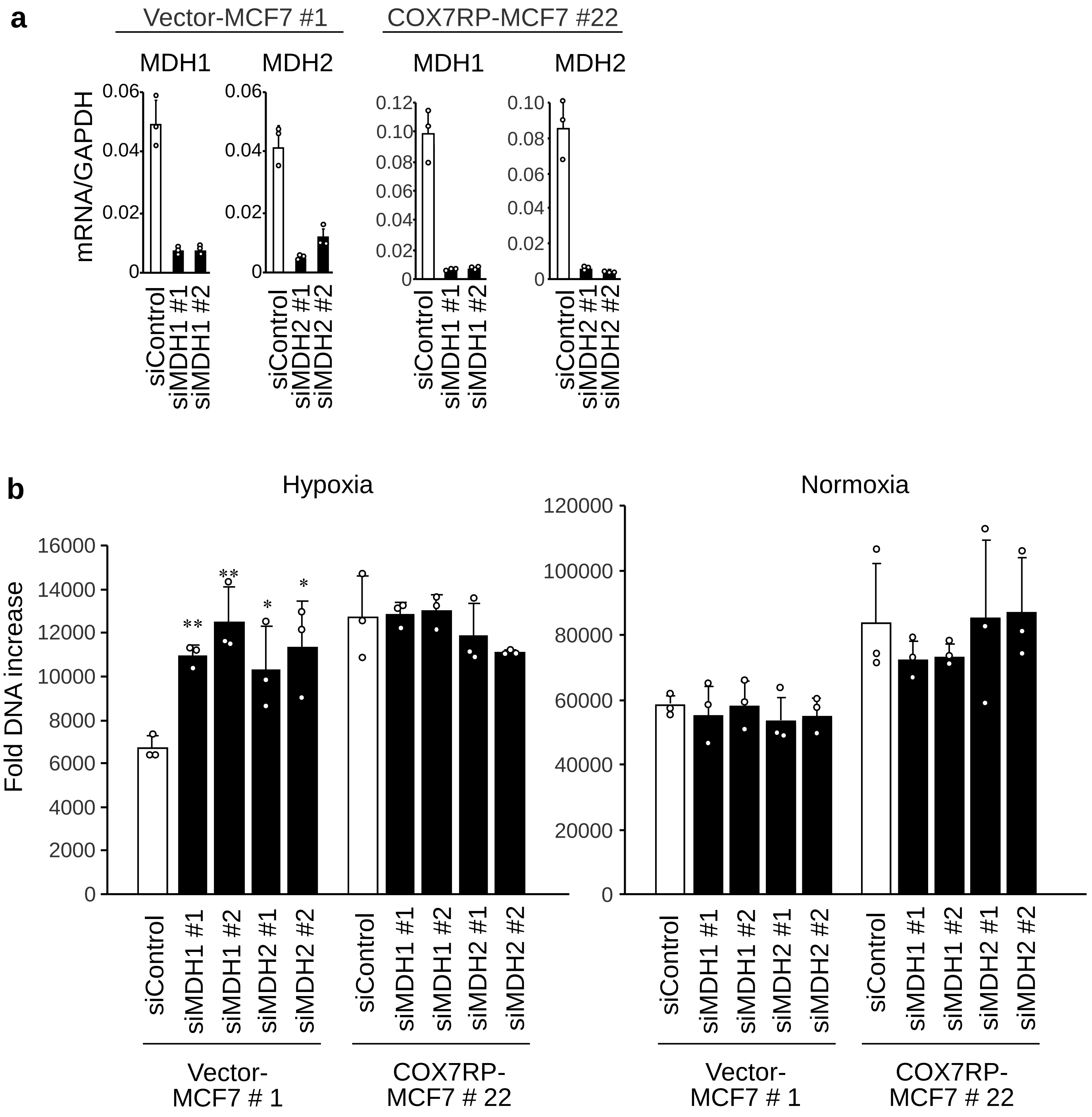


**Supplementary Figure 17 | Silencing of malate dehydrogenase in MCF7 cells.** (**a**) Effects of siRNAs on mRNA expression of malate dehydrogenase. siRNAs targeting malate dehydrogenase (*MDH1* and *MDH2*) were transfected into COX7RP-MCF7 and vector-MCF7 cells. Quantitative RT-PCR analysis of mRNA expression was performed. (**b**) Effects of siRNAs on the growth of vector-MCF7 and COX7RP-MCF7 cells under hypoxic (1% $O_2$) or normoxic conditions. Cell growth was estimated by DNA assay. Data are presented as means ± SD ($n$ = 3 independent experiments). *$P$ < 0.05, **$P$ < 0.01, Student's $t$-test. Source data are provided as a Source Data file.

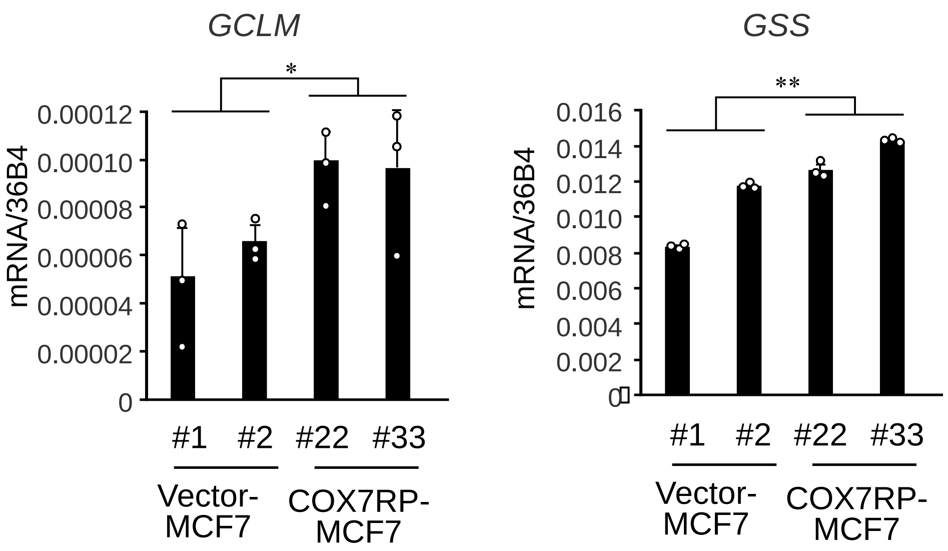


**Supplementary Figure 18 | Glutathione-synthesizing and –modulating enzyme mRNA levels in MCF7 stable transfectants.** Expression levels of glutamate-cysteine ligase modifier subunit (*GCLM*) and glutathione synthetase (*GSS*) were quantified by qRT-PCR analysis. Data are presented as means ± SD ($n$ = 3 independent experiments). *, $P < 0.05$; **, $P < 0.01$, Two-way analysis of variance. Source data are provided as a Source Data file.

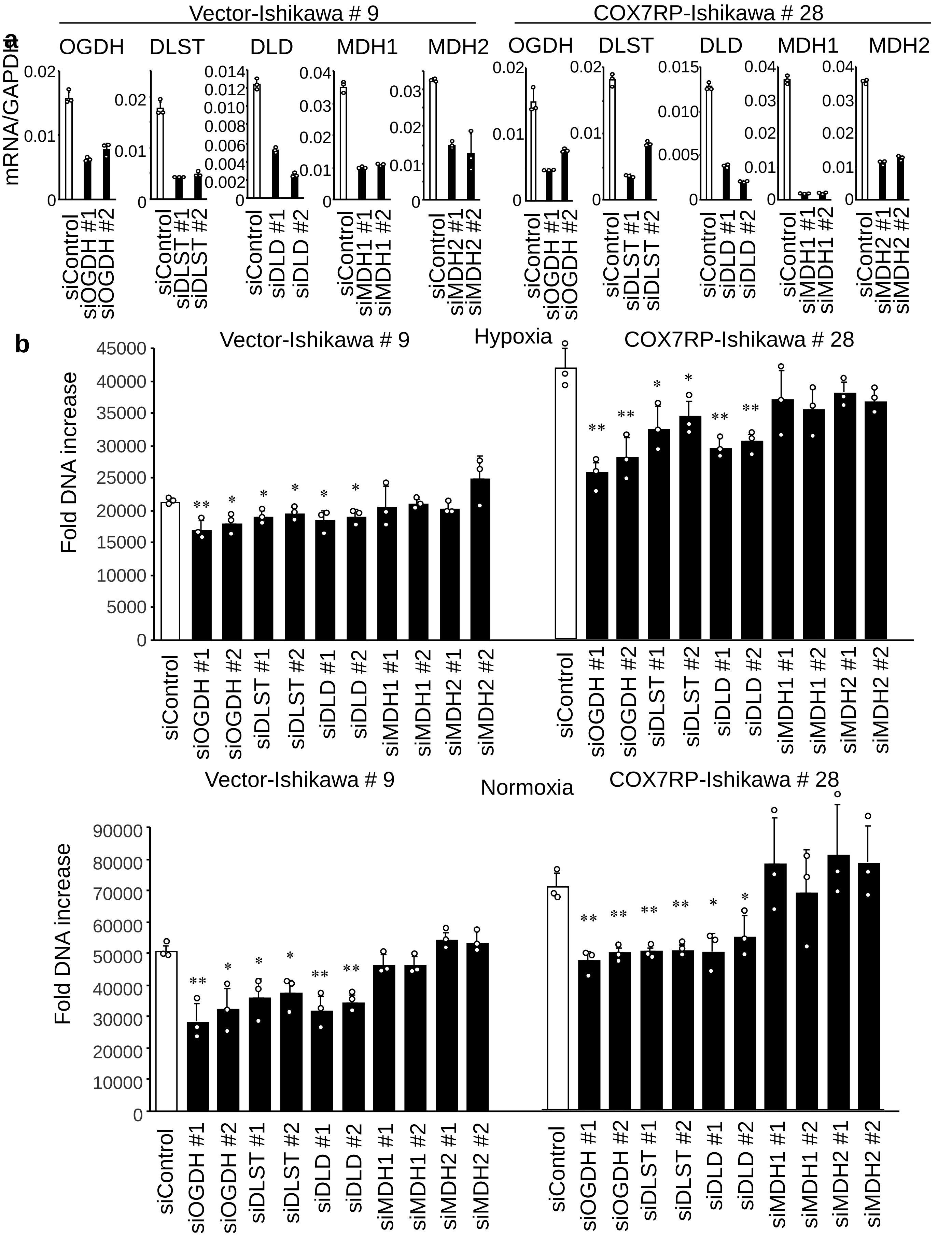


**Supplementary Fig. 19 | Silencing of 2-oxoglutarate dehydrogenase suppresses the growth of Ishikawa cells.** (**a**) Effects of siRNAs on mRNA expression of TCA cycle-related genes. siRNAs targeting each subunit of the 2-oxoglutarate dehydrogenase complex (*OGDH*, *DLST*, and *DLD*) and malate dehydrogenase (*MDH1* and *MDH2*) were transfected into COX7RP-Ishikawa and vector-Ishikawa cells. (**b**) Effects of siRNAs on the growth of vector-Ishikawa and COX7RP-Ishikawa cells under hypoxic or normoxic conditions. Data are presented as means ± SD ($n$ = 3 independent experiments). *$P < 0.05$, **$P < 0.01$, Student's $t$-test. Source data are provided as a Source Data file.

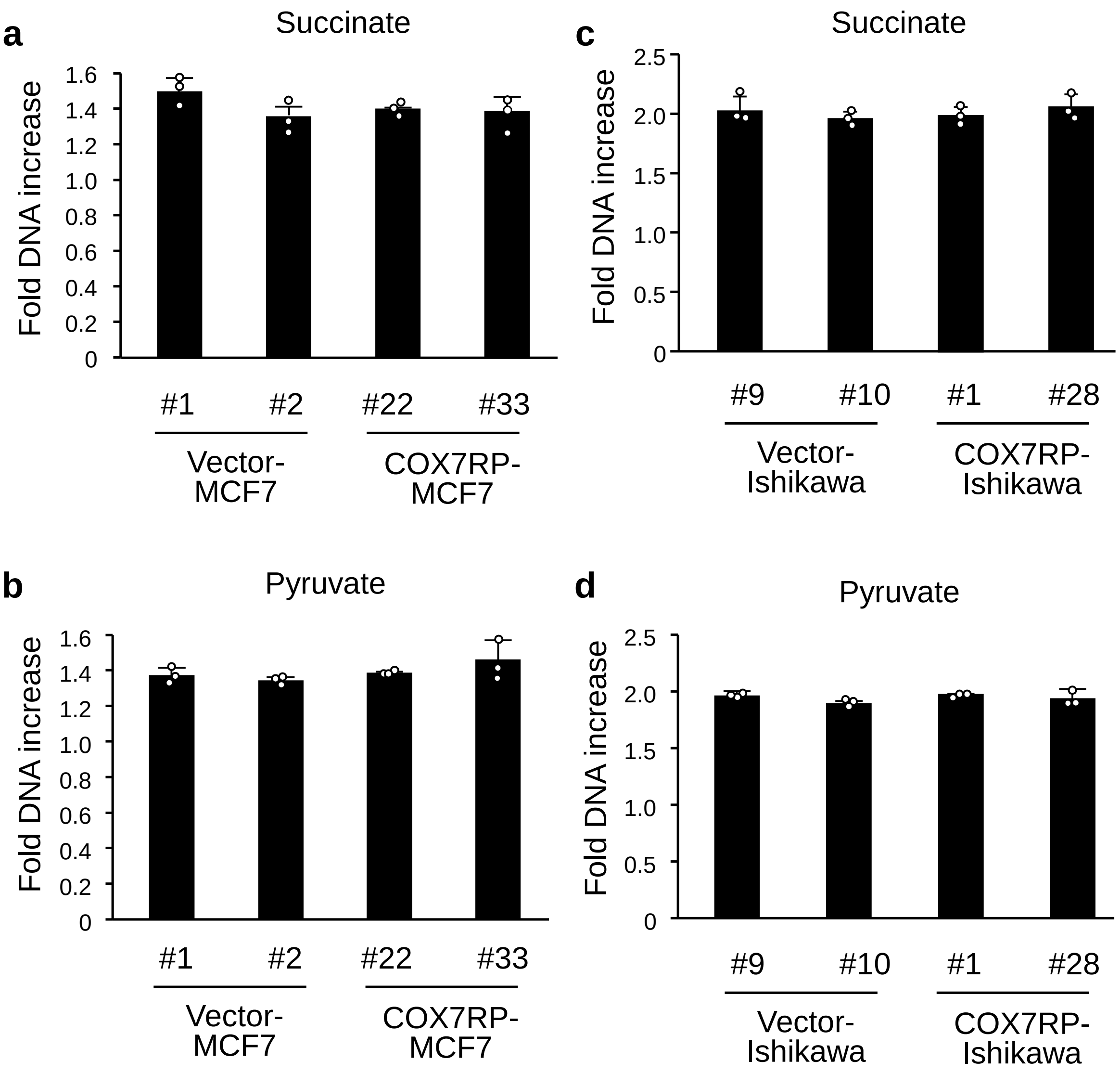


**Supplementary Figure 20 | Effects of pyruvate and succinate supplementation on proliferation of MCF7 stable transfectants under hypoxic condition.** (**a**) Effects of cell-permeable diethylsuccinate (10 mM) on MCF7 transfectant growth in hypoxia 4 days after supplementation. (**b**) Effects of pyruvate (2.5 mM) on MCF7 transfectant growth in hypoxia 4 days after supplementation. (**c**) Effects of cell-permeable diethylsuccinate (10 mM) on Ishikawa transfectant growth in hypoxia 4 days after supplementation. (**d**) Effects of pyruvate (2.5 mM) on Ishikawa transfectant growth in hypoxia 4 days after supplementation. Cell growth was estimated by DNA assay. Data are presented as means ± SD (*n* = 3 independent experiments). Source data are provided as a Source Data file.

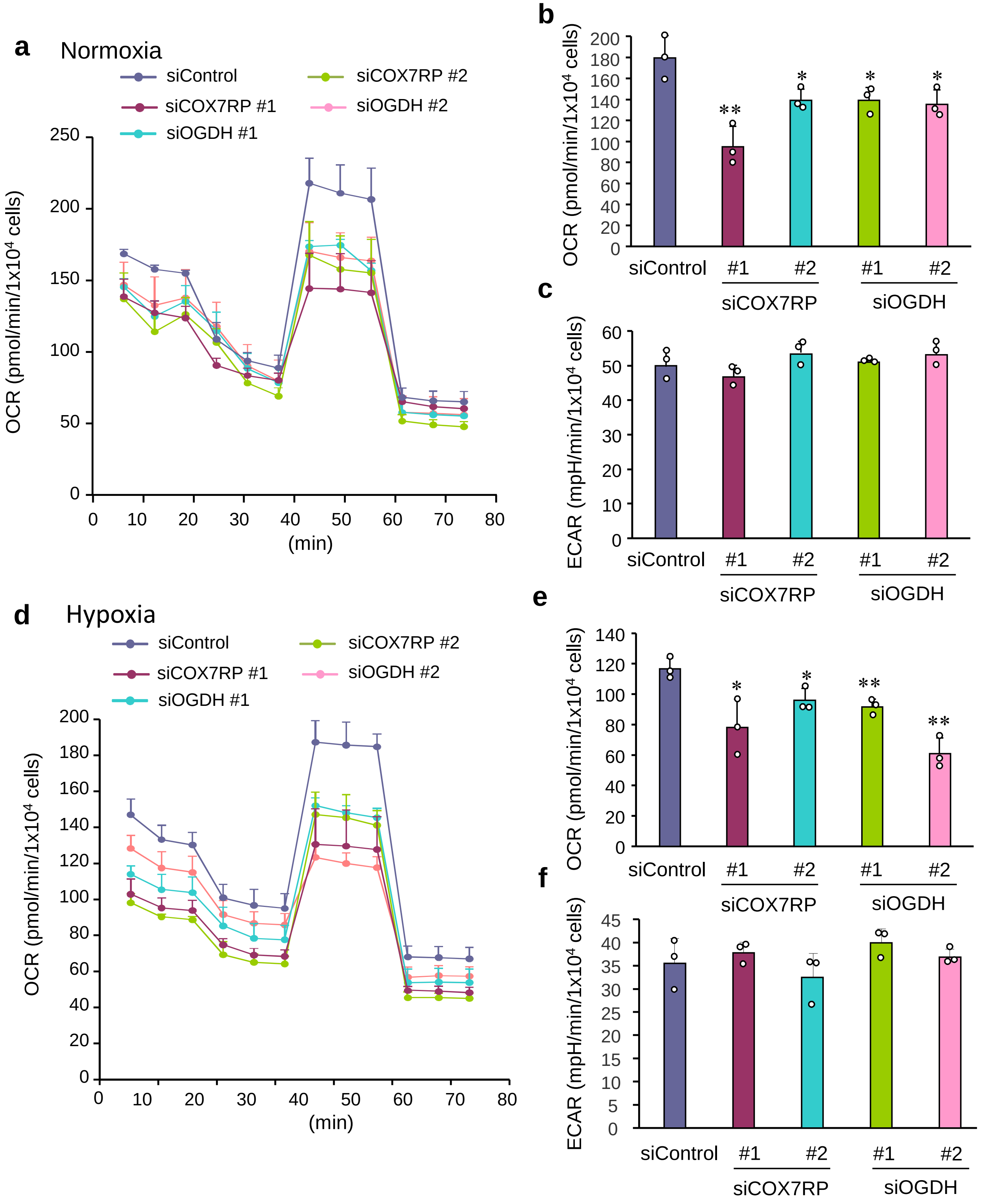


**Supplementary Fig. 21 | Effects of COX7RP and OGDH knockdown on mitochondrial respiration in MCF7 stable transfectants.** (**a**-**c**) Effects of siRNAs targeting OGDH (siOGDH) and COX7RP (siCOX7RP) on OCR and ECAR in normoxia. COX7RP-MCF7 and vector-MCF7 cells were transfected with siOGDHs, siCOX7RPs, and siControl for 48 h. OCRs (a), maximal OCRs (b), and basal ECARs (c) were determined using Seahorse Bioscience XF Extracellular Flux Analyzer with a Cell Mito Stress Test Kit. (**d**-**f**) Effects of siOGDHs and siCOX7RPs on OCR and ECAR in hypoxia. OCRs (d), maximal OCRs (e), and basal ECARs (f) were determined as above. Data were presented as means ± SD ($n$ = 3 biologically independent samples). *$P$ < 0.05, **$P$ < 0.01, Student's $t$-test. Source data are provided as a Source Data file.
