# Supplementary material for: Mitochondrial supercomplex assembly promotes breast and endometrial tumorigenesis by metabolic alterations and enhanced hypoxia tolerance
Source: Nat Commun. 2019 Sep 11;10:4108. doi: 10.1038/s41467-019-12124-6 (PMC6739376; doi:10.1038/s41467-019-12124-6)
Supplement: Supplementary file 2 — Description of Additional Supplementary Files [file 41467_2019_12124_MOESM2_ESM.docx]

**Title: Supplementary Data 1.**
**Description:** Metabolomic profiles of COX7RP transformants of MCF7 and Ishikawa cells in normoxia or hypoxia. Steady-state levels of 116 metabolites were analyzed in COX7RPMCF7 #22, vector-MCF7 #1, COX7RP-Ishikawa #28, and vector-Ishikawa #9 cells by capillary electrophoresis time-of-flight mass spectrometry (CE-TOFMS).

**Title: Supplementary Data 2.**
**Description:** Tracer experiment with [U13C]-labeled glutamine in COX7RP-MCF7 #22 and vector-MCF7 #1 cells in normoxia or hypoxia. Cells were cultured in [U13C]-labeled glutamine for 24 h and the extracts were analyzed by CE-TOFMS.
