# Supplementary material for: Mitochondrial supercomplex assembly promotes breast and endometrial tumorigenesis by metabolic alterations and enhanced hypoxia tolerance
Source: Nat Commun. 2019 Sep 11;10:4108. doi: 10.1038/s41467-019-12124-6 (PMC6739376; doi:10.1038/s41467-019-12124-6)
Supplement: Supplementary file 5 — Reporting Summary [file 41467_2019_12124_MOESM5_ESM.pdf]

nature research

Corresponding author(s): Satoshi Inoue

Last updated by author(s): Aug 3, 2019

Reporting Summary

Nature Research wishes to improve the reproducibility of the work that we publish. This form provides structure for consistency and transparency in reporting. For further information on Nature Research policies, see Authors & Referees and the Editorial Policy Checklist.

Statistics

For all statistical analyses, confirm that the following items are present in the figure legend, table legend, main text, or Methods section.

n/a	Confirmed	
☐	☒	The exact sample size (*n*) for each experimental group/condition, given as a discrete number and unit of measurement
☐	☒	A statement on whether measurements were taken from distinct samples or whether the same sample was measured repeatedly
☐	☒	The statistical test(s) used AND whether they are one- or two-sided<br>*Only common tests should be described solely by name; describe more complex techniques in the Methods section.*
☐	☒	A description of all covariates tested
☐	☒	A description of any assumptions or corrections, such as tests of normality and adjustment for multiple comparisons
☐	☒	A full description of the statistical parameters including central tendency (e.g. means) or other basic estimates (e.g. regression coefficient) AND variation (e.g. standard deviation) or associated estimates of uncertainty (e.g. confidence intervals)
☒	☐	For null hypothesis testing, the test statistic (e.g. *F*, *t*, *r*) with confidence intervals, effect sizes, degrees of freedom and *P* value noted<br>*Give P values as exact values whenever suitable.*
☒	☐	For Bayesian analysis, information on the choice of priors and Markov chain Monte Carlo settings
☒	☐	For hierarchical and complex designs, identification of the appropriate level for tests and full reporting of outcomes
☒	☐	Estimates of effect sizes (e.g. Cohen's *d*, Pearson's *r*), indicating how they were calculated

Our web collection on statistics for biologists contains articles on many of the points above.

Software and code

Policy information about availability of computer code

Data collection	No software was used.
Data analysis	Excel (version 2013) and ImageJ (version 1.50b) were used.

For manuscripts utilizing custom algorithms or software that are central to the research but not yet described in published literature, software must be made available to editors/reviewers. We strongly encourage code deposition in a community repository (e.g. GitHub). See the Nature Research guidelines for submitting code & software for further information.

Data

Policy information about availability of data

All manuscripts must include a data availability statement. This statement should provide the following information, where applicable:

- Accession codes, unique identifiers, or web links for publicly available datasets
- A list of figures that have associated raw data
- A description of any restrictions on data availability

The accession number for microarray data reported in Supplementary Table 8 is GSE124524. Data of metabolomic analysis and tracer experiment performed in this study are provided by Supplementary Data 1 and 2, respectively. The source data underlying Figs. 2-8, Supplementary Tables 3-7, and Supplementary Figs. 2-14 and 16-21 are provided as a Source Data file. All the other data supporting the findings of this study are available from the corresponding authors upon reasonable request.

Field-specific reporting

Please select the one below that is the best fit for your research. If you are not sure, read the appropriate sections before making your selection.

☒ Life sciences ☐ Behavioural & social sciences ☐ Ecological, evolutionary & environmental sciences

For a reference copy of the document with all sections, see nature.com/documents/nr-reporting-summary-flat.pdf

Life sciences study design

All studies must disclose on these points even when the disclosure is negative.

Sample size	No sample-size calculation was performed.
Data exclusions	No data were excluded.
Replication	All attempts at replication were successful.
Randomization	All allocation was random.
Blinding	The investigators were blinded to group allocation.

Reporting for specific materials, systems and methods

We require information from authors about some types of materials, experimental systems and methods used in many studies. Here, indicate whether each material, system or method listed is relevant to your study. If you are not sure if a list item applies to your research, read the appropriate section before selecting a response.

Materials & experimental systems

n/a	Involved in the study	
☐	☒	Antibodies
☐	☒	Eukaryotic cell lines
☒	☐	Palaeontology
☐	☒	Animals and other organisms
☐	☒	Human research participants
☒	☐	Clinical data

Methods

n/a	Involved in the study	
☒	☐	ChIP-seq
☒	☐	Flow cytometry
☒	☐	MRI-based neuroimaging

Antibodies

Antibodies used	RISP (ab14746, Abcam), COX1 (ab14705, Abcam), COX4 (4844, Cell signaling), β-actin (AC-74, Sigma-Aldrich), NDUFA9 (459100, Invitrogen), Fp70 (459200, Invitrogen), and COX7RP (Ikeda et al., Nat Commun. 4, 2147, 2013)
Validation	All antibodies can detect human proteins.

Eukaryotic cell lines

Policy information about cell lines

Cell line source(s)	Ishikawa cells were kindly provided by Dr. Masato Nishida (Kasumigaura Medical Center, Ibaraki, Japan). MCF7 and 293T cells were obtained from American Type Culture Collection (Rockville, MD, USA).
Authentication	Authentication of cell lines were done by STR analysis at the outside laboratory.
Mycoplasma contamination	All cell lines were negative for Mycoplasma contamination.
Commonly misidentified lines (See ICLAC register)	None of the cell limes used in this study is present in the database of commonly misidentified lines.

Animals and other organisms

Policy information about studies involving animals; ARRIVE guidelines recommended for reporting animal research

Laboratory animals	Female athymic mice (8-week-old BALB/c nu/nu)

Wild animals	This study did not use wild animals.
Field-collected samples	This study did not use materials collected from field.
Ethics oversight	All animal experiments were approved by the Animal Care and Use Committee of Saitama Medical University and conducted in accordance with the Guidelines and Regulations for the Care and Use of Experimental Animals by Saitama Medical University.

Note that full information on the approval of the study protocol must also be provided in the manuscript.

Human research participants

Policy information about studies involving human research participants

Population characteristics	Woman, diagnosed with breast cancer or endometrial cancer
Recruitment	Patients were recruited into study randamly.
Ethics oversight	The ethics committee of Tohoku University School of Medicine approved the research protocols and informed consent was obtained from these patients before surgery in the institution.

Note that full information on the approval of the study protocol must also be provided in the manuscript.
